# Environmental Outcomes of Reducing Medication Waste by Redispensing Unused Oral Anticancer Drugs

**DOI:** 10.1001/jamanetworkopen.2024.38677

**Published:** 2024-10-10

**Authors:** Elisabeth M. Smale, Anne B. Ottenbros, Bart J. F. van den Bemt, Eibert R. Heerdink, Jelle Verploegen, Rosalie van Zelm, Toine C. G. Egberts, Charlotte L. Bekker

**Affiliations:** 1 Department of Pharmacy, Radboud University Medical Center, Nijmegen, the Netherlands; 2 Department of Environmental Science, Radboud University, Nijmegen, the Netherlands; 3 Department of Pharmacy, Sint Maartenskliniek, Ubbergen, the Netherlands; 4 Division of Laboratory, Genetics and Pharmacy, Department of Clinical Pharmacy, University Medical Centre Utrecht, Utrecht, the Netherlands; 5 Faculty of Science, Utrecht Institute for Pharmaceutical Sciences, Division of Pharmacoepidemiology and Clinical Pharmacology, Utrecht University, Utrecht, the Netherlands; 6 Utrecht University of Applied Sciences, Medical Centre Utrecht, Research Group Innovations of Pharmaceutical Care, Utrecht, the Netherlands

## Abstract

**Importance:**

Medications are associated with substantial environmental outcomes, yet frequently end up being unused by patients. Waste-minimizing interventions, such as redispensing of quality-approved oral anticancer drugs remaining unused by patients at home, could reduce the environmental footprint of cancer treatment.

**Objectives:**

To assess the environmental outcomes of redispensing quality-assured oral anticancer drugs and to explore how redispensing could be environmentally optimized.

**Design, Setting, and Participants:**

In this quality improvement study, a cradle-to-grave life cycle assessment was performed in the outpatient pharmacy of 4 Dutch hospitals, based on a prospective multicenter trial comprising 1071 patients with a clinical diagnosis of cancer and an active prescription for an oral anticancer drug stored at room temperature from February 1, 2021, to February 1, 2023, with a follow-up of 12 months per patient.

**Intervention:**

Participants received prescribed oral anticancer drugs with additional quality-assurance materials (ie, seal bags and time-temperature indicators), so the pharmacy could redispense quality-assured drugs based on authenticity, appearance, remaining shelf life, and/or adequate storage.

**Main Outcomes and Measures:**

The estimated environmental outcomes avoided due to waste reduction (ie, production and transport and incineration of redispensed oral anticancer drugs) corrected for outcomes of process burdens (ie, quality assurance materials), quantified in 3 outcome measures: human health damage (disability-adjusted life-years), ecosystems damage (species × year), and climate change (kg of carbon dioxide equivalent [CO_2-eq_]) per patient per year.

**Results:**

A volunteer sample of 1071 patients (median age, 70 years [IQR, 62-75 years]; 622 men [58.1%]) participated in the intervention. Redispensing oral anticancer drugs was initially associated with an environmental burden, mainly because of the high impact of time-temperature indicators. However, when quality-assurance materials were selectively used for temperature-sensitive oral anticancer drugs (ie, maximum storage temperature of 25 °C), redispensing was environmentally beneficial to human health and ecosystems, providing estimated climate benefits of 1.9 kg (95% CI, 1.4-2.6 kg) of CO_2-eq_ per patient per year.

**Conclusions and Relevance:**

In this quality improvement study, redispensing unused oral anticancer drugs was found to be a suitable strategy to reduce waste and improve environmental sustainability of cancer treatment after process optimization. Redispensing unused oral anticancer drugs could contribute to sustainability of cancer treatment through reduced costs and environmental outcomes.

## Key Points

**Question:**

When is redispensing quality-assured oral anticancer drugs that remained unused by patients environmentally beneficial compared with the standard practice of disposal?

**Findings:**

This quality improvement study found that redispensing unused oral anticancer drugs was associated with environmental benefits if quality assurance materials were selectively used for temperature-sensitive drugs, providing climate benefits of 1.9 kg (95% CI, 1.4-2.6 kg) of carbon dioxide equivalent per patient per year.

**Meaning:**

This study suggests that redispensing unused oral anticancer drugs could increase the environmental sustainability of cancer treatment.

## Teaser

This quality improvement study assesses the environmental outcomes of redispensing quality-assured oral anticancer drugs compared with the standard practice of disposal and examines how redispensing could be environmentally optimized.

## Introduction

Medication is intended to improve health, yet can pose a threat to planetary health through its substantial environmental impacts. Medication is a main source of the greenhouse gas emissions produced by health care and contributes substantially to other environmental indicators, such as material extraction, water consumption, and land use.^1,2^ Pharmaceutical manufacturing is a resource-intensive industry associated with global emissions of 52 metric tons of carbon dioxide equivalent (CO_2-eq_) in 2015.^3^ Any waste of medication therefore equates to unnecessary resource consumption and emissions, while it also is associated with additional negative environmental outcomes through incineration. Disposal of unused medications also underscores the challenge of ensuring sustainable and affordable access to expensive drugs, such as oral anticancer drugs (OADs).^4^

Oral anticancer drugs frequently remain unused by patients, resulting in the disposal of valuable resources, due to, for instance, insufficient effect or adverse effects.^5,6^ Waste prevention is the most desirable strategy to conserve resources. Accordingly, up to one-third of OAD waste could be prevented.^7,8^ To further minimize waste, additional strategies have been explored, such as the redispensing of unused medication.^9^ In this strategy, medication that remained unused by patients is collected by a pharmacy to be redispensed to other patients instead of merely collected for incineration (ie, standard practice). Redispensing unused medication is not widely implemented due to quality concerns related to unknown storage conditions in patients’ homes, resulting in legal restrictions.^10^ Pharmaceutical quality could, however, be ensured through a quality assurance procedure with, for instance, temperature-sensing technologies and sealed packaging.^11,12,13,14^

A recent large-scale multicenter trial showed that redispensing unused OADs reduced waste by two-thirds and that substantial cost savings per patient were achieved.^9^ It was hypothesized that redispensing unused OADs is also environmentally beneficial because fewer OADs have to be produced, transported, or incinerated. However, there is not much information on the environmental outcomes of cancer medication production, and thus it remains unclear whether the avoided environmental outcomes associated with waste reduction compensate for the impacts of the materials used in the quality assurance procedure. Such insights can provide targets for waste minimization as well as implementation of more sustainable care practices. Therefore, this study assessed the environmental outcomes of redispensing quality-assured OADs and examined how redispensing could be optimized from an environmental perspective.

## Methods

### Redispensing Program

**Figure 1.**
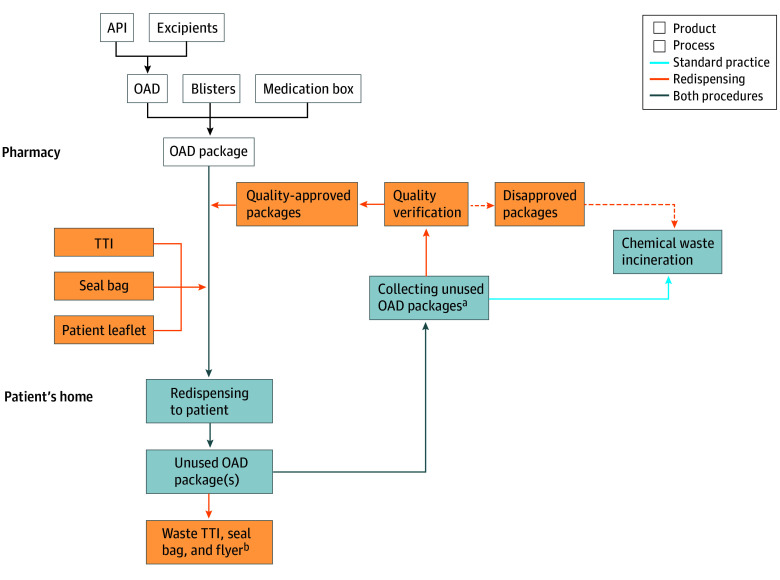
Overview of Redispensing Unused Oral Anticancer Drugs (OADs), Standard Practice, and Overlapping Procedures and OAD Package Production The arrows encompass transport steps. API indicates active pharmaceutical ingredient; TTI, time-temperature indicator. ^a^A total of 2.6% of dispensed OAD packages (335 of 13 069) were returned to the pharmacy during the trial. ^b^Patients were requested to return TTIs for recycling (60% was recycled) and dispose of the seal bag and patient leaflet through regular disposal routes for plastic and paper recycling.

This quality improvement study consisted of an environmental outcome analysis based on a redispensing program that was evaluated in a prospective single-group trial (ROAD [Redispensing Oral Anticancer Drugs] study; World Heatlh Organization International Clinical Trials Registry Platform [ICTRP] Identifier: NL9208), which has previously been described in detail.^9^ Patients with a diagnosis of cancer and treated with OADs (eTable 1 in Supplement 2) in 4 Dutch hospitals were eligible for inclusion between February 1, 2021, and February 1, 2023, and were followed up for a period up to 1 year. During the trial, patients received prescribed OADs with a time-temperature indicator (TTI) in sealed packaging, to ensure drug quality and authenticity on redispensing (Figure 1). In total, 171 of 1071 patients (16.0%) returned 335 unused OAD packages of 13 069 packages dispensed (2.6%) during regular hospital visits, of which 228 (68.1%) were of approved quality and redispensed. Pharmaceutical quality was checked by the outpatient pharmacy, based on (1) enclosed sealed packaging, (2) undamaged outer packaging, (3) remaining shelf life of 6 months or more, and (4) storage temperature corresponding to the Summary of Product Characteristics, measured with a TTI. Oral anticancer drugs of approved quality were redispensed to other patients prescribed the same OAD (Figure 1). Ethical review was waived by the local medical research ethics committee of the Radboud University Medical Center (METC Oost Nederland), and the protocol was previously published.^9^ All patients provided written informed consent, and the trial complied with the Declaration of Helsinki.^15^ This study followed the ISO14040 standards and harmonized rules for life cycle assessments (LCAs) on pharmaceutical products and processes^16,17^ and the Standards for Quality Improvement Reporting Excellence (SQUIRE) reporting guideline for reporting quality improvement studies.^18^

### Life Cycle Assessment

#### Scope of Study

A cradle-to-grave LCA was performed to assess the environmental outcomes of redispensing unused OAD packages (Figure 1). Currently, standard practice is the disposal of unused OADs through incineration; thus, by redispensing, the environmental outcomes of producing, transporting, and incinerating OAD packages were avoided. Nevertheless, the redispensing procedure is accompanied by its own environmental outcomes, relating to quality assurance materials (ie, TTI, seal bag, and patient information leaflet) (eMethods in Supplement 2). Environmental outcomes were assessed per functional unit of treating 1 patient with OADs for 1 year. Patients were requested to return unused OADs during regular hospital visits to prevent additional transport or disposal at home.

#### Data Collection

##### Quality Assurance Procedure Materials

Quality assurance materials (ie, patient leaflet, seal bag, and TTI) were separated into components containing single materials and weighed. Background data on these components were taken from ecoinvent, version 3.8 (ecoinvent).^19^ It was assumed that paper (from the patient leaflet and secondary packaging materials) and plastic (from the seal bag) were recycled, following Dutch waste management protocols.^20^ The TTIs were collected in the pharmacy for reuse, and, if reuse was not possible, batteries were recycled. In total, 59.4% of TTIs (7768 of 130 69) were reused at least once.

##### Oral Anticancer Drugs

A sample of 6 OADs was selected to calculate the estimates of the environmental outcomes of all redispensed OADs: hydroxycarbamide, lenalidomide, olaparib, pomalidomide, sunitinib, and temozolomide. The sample was based on the variety in (1) the molecular complexity of OADs, (2) the therapeutic mechanism of OADs, and (3) the number of redispensed OAD packages.

Inventory data were gathered for production, distribution, and incineration of OAD packages (eMethods in Supplement 2). First, the production processes of the active pharmaceutical ingredients (APIs) were modeled based on chemical reaction pathways found via Reaxys.^21^ All modeled chemical reactions were scaled up to industrial scale,^22^ and background processes were taken from ecoinvent, version 3.8.^19^ The second step encompassed galenic formulation: the addition of excipients to APIs and the mixing processes to create unit doses (ie, tablets or capsules).^23^ Third, medication packaging processes, consisting of primary packaging material (ie, blisters or jars) and secondary packaging material (ie, Summary of Product Characteristics and carton box), were modeled. The transportation between manufacturing sides, wholesalers, and pharmacies were included. Disposal of OADs and other waste was assumed to take place in the Netherlands through incineration as chemical hazardous waste.

#### Environmental Outcome Assessment

Life cycle assessment results were calculated with the outcome assessment method ReCiPe 2016 (H), version 1.07, in SimaPro, version 9.4.0.2 (Pré Consultants bv).^24^ The study focused on 2 environmental areas of protection (ie, end points): damage to human health and damage to ecosystems. Damage to human health indicates the association of emissions and resource extraction with human health in disability-adjusted loss of years for a person (ie, disability-adjusted life-years [DALYs]), and damage to ecosystems is the loss of integrated species over time (species × year). In addition, this study reported the environmental outcome of climate change expressed in kilograms of carbon dioxide equivalent (CO_2-eq_), as this allows for comparison with other pharmaceutical LCA studies.^16^ A contribution analysis was used to identify the primary environmental issues contributing most to the environmental end points.

IBM SPSS Statistics for Windows, version 25 (IBM Corp) was used to calculate mean environmental outcomes per patient per year, using bootstrapping (n = 2000). Proportions were displayed with 95% CIs to demonstrate statistical variability within the data obtained from the trial.

#### Quality Assurance Procedure Scenarios

The least controversial quality assurance procedure was used to ensure patient safety in the trial (ie, base case quality assurance procedure). In addition, 2 downscaled quality assurance procedure scenarios were explored to assess the potential for optimizing the environmental outcomes of redispensing unused OADs (Table).^9^ Because no temperature breaches exceeding 30 °C were reported in the trial and a shelf-life criterion of 2 months provides sufficient time for redispensing,^9^ clinical guidelines advise to monitor temperature for medication only with a maximum storage temperature of 25 °C and to use a reduced shelf-life criterion,^25^ referred to as the optimized quality assurance procedure. In addition, a visual quality assurance procedure scenario was investigated, as existing redispensing programs for the purpose of medication donations monitor medication quality based on visual checks.^26,27^

**Table.** Redispensing Scenarios and Percentage of OAD Packages Redispensed per Quality Assurance Procedure Scenario

Quality assurance procedure		Redispensed OAD packages^a^	
Scenario	Criteria	No.	% (95% CI)
Base case	(1) Sealed packaging and undamaged outer packaging (2) Shelf life ≥6 mo (3) Temperature monitoring of all OADs (60% TTIs reused)	228	1.7 (1.6-1.9)
Optimized	(1) Undamaged outer packaging (2) Shelf life ≥2 mo (3) Temperature monitoring for OADs with maximum storage temperature of 25 °C (60% TTIs reused)	250	1.9 (1.8-2.0)
Visual	(1) Shelf life ≤2 mo	335	2.6 (2.4-2.7)^b^

Abbreviations: OAD, oral anticancer drug; TTI, time-temperature indicator.

^a^ In total, 13 069 OAD packages were dispensed to 1071 patients during 1 year. Oral anticancer drug packages contained a mean of 27 daily doses. In all, 171 patients (16.0%) returned 335 unused OAD packages (2.6%).^9^

^b^ All returned OAD packages were redispensed.

### Statistical Analysis

#### Extrapolation to All Redispensed OADs

A sensitivity analysis was performed to study the uncertainty in the extrapolation of the environmental outcomes of the 6 studied OADs to all redispensed OADs. The outcome per OAD package was assessed by the number of unit doses (ie, tablet or capsule) per package and the dosage of API per unit dose. Extrapolation was based on the mean environmental outcomes for API production found for the 6 studied OADs, the lowest outcomes and the highest outcomes, combined with the OAD-specific dosage and number of unit doses (eTable 3 in Supplement 2).

#### Contribution Analyses

Contribution analyses were performed to verify avoidance of burden shifting in the selected outcome categories, on the quality assurance materials, per OAD package and per gram of API production, to gain insight into the environmental hotspots of materials and subsequently guide potential improvement in the redispensing program.

## Results

### Study Population

After screening 2782 patients for eligibility, 2426 patients were invited to participate in the redispensing program. In total, 1223 patients consented to participate, of whom 1071 (median age, 70 years [IQR, 62-75 years]; 622 men [58.1%] and 449 women [41.9%]) participated in the redispensing program. Participants used targeted therapies (56.8% [655 of 1071]), cytotoxic agents (22.1% [255 of 1071]), endocrine therapy (13.2% [152 of 1071]), and/or immunosuppressants (7.9% [91 of 1071]) at study entry for solid cancers (51.2% [548 of 1071]) or hematologic cancers (48.8% [523 of 1071]). Treatment onset with OADs was 6 months or less (32.4% [347 of 1071]), 7 to 24 months (31.6% [338 of 1071]), or more than 24 months (36.0% [386 of 1071]) at inclusion, with 60 of 1071 patients (5.6%) initiating a new OAD treatment and 305 of 1071 patients (28.1%) discontinuing OAD treatment during the study period. The study sample and economic costs of treatment have previously been described in more detail.^9^

### Environmental Outcomes of Redispensing Unused OADs

**Figure 2.**
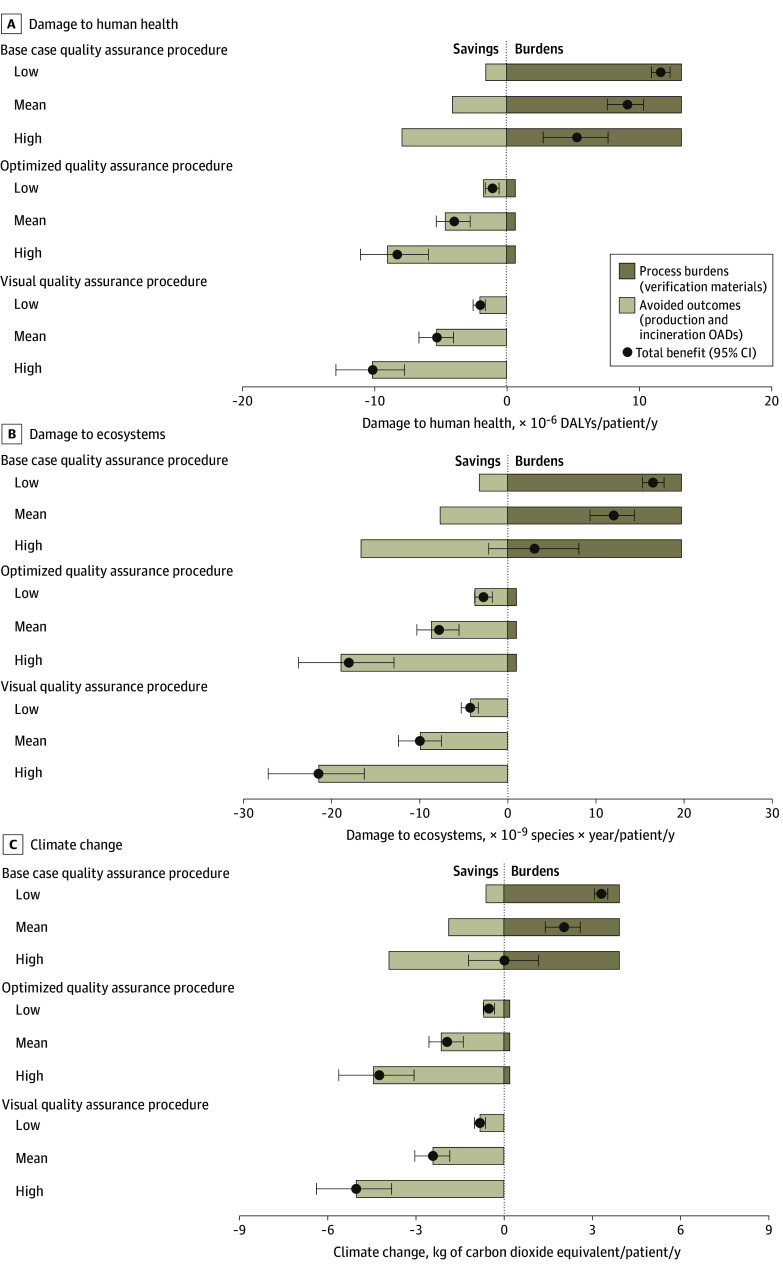
Environmental Outcomes of Redispensing 3 Different Quality Assurance Scenarios A, Damage to human health. B, Damage to ecosystems. C, Climate change. DALY indicates disability-adjusted life-year; OAD, oral anticancer drug.

To obtain environmental benefits, the avoided environmental outcomes of OAD production and incineration must outweigh the processing outcomes of quality assurance. For the base case quality assurance procedure, redispensing was not associated with environmental benefits (Figure 2). By redispensing unused OADs, the negative environmental outcomes of OAD production, transport, and incineration were avoided of 4.1 × 10^–6^ (95% CI, 2.9 × 10^–6^ to 5.3 × 10^–6^) DALYs, 7.7 × 10^–9^ (95% CI, 5.5 × 10^–9^ to 1.0 × 10^–8^) species × year, and 1.9 kg (95% CI, 1.3-2.4 kg) of CO_2-eq_ per patient per year with the base case quality assurance procedure (Figure 2). However, these avoided outcomes did not outweigh the process burden of the base case quality assurance procedure of 1.3 × 10^–5^ (95% CI, 1.3 × 10^–5^ to 1.4 × 10^–5^) DALYs, 2.0 × 10^–8^ (95% CI, 1.9 × 10^–8^ to 2.1 × 10^–8^) species × year, and 3.9 kg (95% CI, 3.8-4.1 kg) of CO_2-eq_ per patient per year. In total, the base case quality assurance procedure resulted in outcomes of 9.1 × 10^–6^ (95% CI, 7.6 × 10^–6^ to 1.0 × 10^–5^) DALYs, 1.2 × 10^–8^ (95% CI, 9.4 × 10^–9^ to 1.4 × 10^–8^) species × year, and 2.1 kg (95% CI, 1.4-2.6 kg) of CO_2-eq_ per patient per year (eAppendix in Supplement 1).

### Quality Assurance Procedure Scenarios

Different quality assurance scenarios showed the environmental benefits associated with redispensing unused OADs for all environmental indicators (Figure 2). For both scenarios, a higher number of unused OADs could be redispensed (Table).^9^ In addition, outcomes of the quality assurance were reduced by 94.9% for human health, 95.3% for ecosystems, and 94.9% for climate change in the optimized quality assurance procedure and were zero when only visual checks were used. This was associated with total environmental benefits of 4.0 × 10^–6^ (95% CI, 2.8 × 10^–6^ to 5.3 × 10^–6^) DALYs, 7.8 × 10^–9^ (95% CI, 5.5 × 10^–9^ to 1.0 × 10^–8^) species × year, and 1.9 kg (95% CI, 1.4-2.6 kg) of CO_2-eq_ per patient per year in the optimized quality assurance procedure and 5.3 × 10^–6^ (95% CI, 4.0 × 10^–6^ to 6.6 × 10^–6^) DALYs, 9.9 × 10^–9^ (95% CI, 7.5 × 10^–9^ to 1.2 × 10^–8^) species × year, and 2.4 kg (95% CI, 1.8-3.0 kg) of CO_2-eq_ per patient per year for visual quality checks (Figure 2).

### Extrapolation to All Redispensed OADs

**Figure 3.**
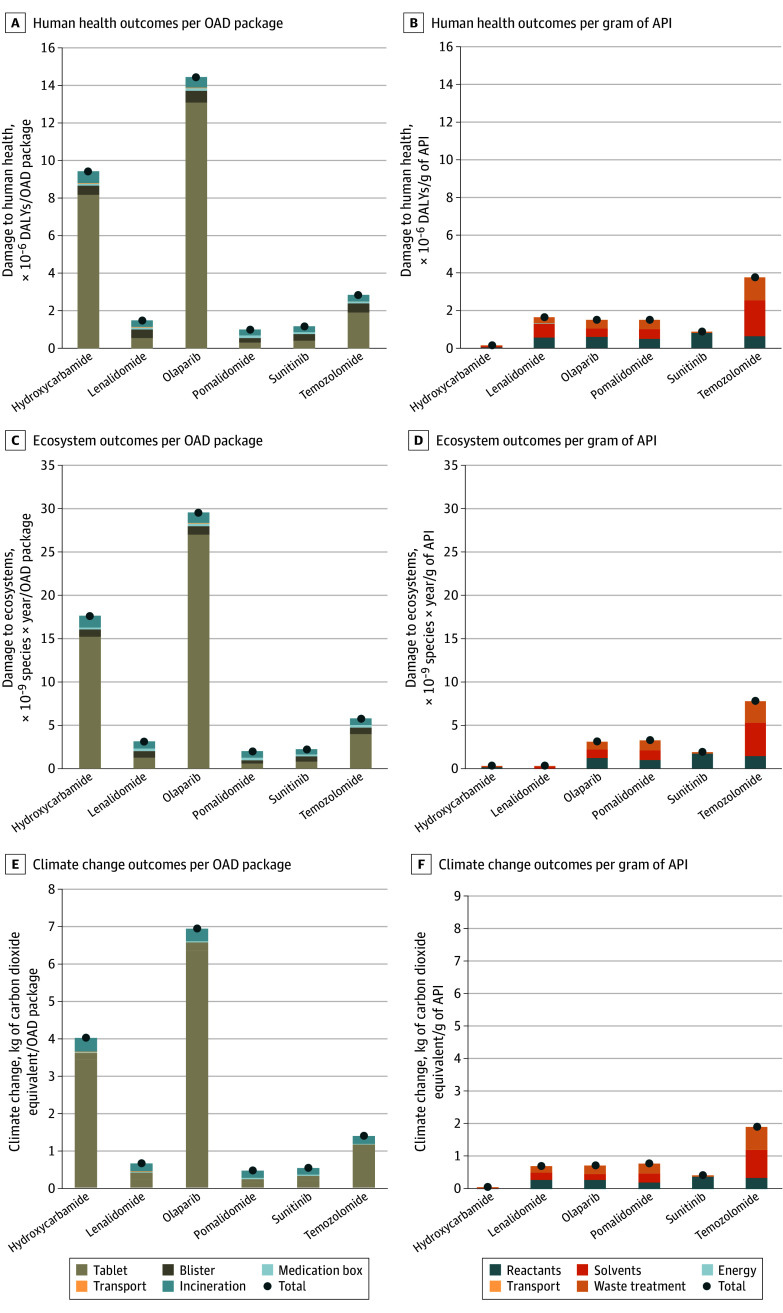
Contribution Analysis of the Avoided Outcomes of Oral Anticancer Drug (OAD) Production and Incineration A, Damage to human health per OAD package. B, Damage to human health per gram of active pharmaceutical ingredient (API). C, Damage to ecosystems per OAD package. D, Damage to ecosystems per gram of API. E, Climate change per OAD package. F, Climate change per gram of API. Each OAD package contains tablets in blisters, summary of product characteristics, and outer medication box. DALY indicates disability-adjusted life-year.

The lowest outcome for API production was found for hydroxycarbamide and the highest for temozolomide (Figure 3). Varying the outcomes of API production still showed unbeneficial (or neutral) outcomes on all indicators for redispensing according to the base case quality assurance procedure, while environmental benefits were associated with both the optimized and visual quality assurance procedures (Figure 2). Specifically, for climate change, using the highest outcomes resulted in equal process burdens and avoided negative outcomes. Production of 1 kg of API ranged from 57.6 of kg CO_2-eq_ for hydroxycarbamide to 1910.9 kg of CO_2-eq_ for temozolomide.

### Contribution Analysis Process Burdens

Time-temperature indicators were the primary contributor to process burdens, resulting in 89.9% of outcomes associated with damage to human health, 91.7% associated with damage to ecosystems, and 86.9% associated with climate change (eFigure 9 in Supplement 2). Reducing the quantity of TTIs used for redispensing, as done in the optimized quality assurance procedure, or eliminating TTIs, as done in the visual checks, lowered process burdens substantially. Closer inspection of the components of TTI production showed that its outcome was associated mainly with small electronic components (ie, resistors and capacitors).

### Avoidance of Negative Outcomes With Contribution Analysis 

Environmental outcomes varied largely between the 6 different OAD packages, ranging from 1.0 × 10^–6^ DALYs, 2.0 × 10^–9^ species × year, and 0.4 kg of CO_2-eq_ per OAD package for pomalidomide to 1.4 × 10^–5^ DALYs, 3.0 × 10^–8^ species × year, and 6.9 of kg CO_2-eq_ per OAD package for olaparib (Figure 3A, C, and E). Negative outcomes were associated mainly with OAD manufacturing, which was dependent on the API dosage per package (ie, the strength and quantity of unit doses per package). For instance, hydroxycarbamide had the smallest environmental outcomes for API production (1.4 × 10^–7^ DALYs per kg of API, 2.6 × 10^–10^ species × year, and 57.6 kg of CO_2-eq_ per gram API), while it had one of the largest outcomes for OAD tablet production (eFigure 10 in Supplement 2). When zooming in on API production (Figure 3B, D, and F), use and waste treatment of solvents and reactants were the main contributors. Transport and energy use during production showed only small contributions.

### Contribution Analysis Outcome Assessment

The analysis of the contribution of single environmental problems to the 2 areas of protection (ie, damage to human health and damage to ecosystems) showed that climate change was the major contributing factor (eFigures 11-13 in Supplement 2).

## Discussion

This study showed that redispensing unused OADs can be environmentally beneficial. Because the TTIs used for safe redispensing were the main factors associated with adverse environmental outcomes, the optimized scenario in which TTIs were used only for temperature-sensitive OADs (ie, maximum storage temperature of 25 °C) was associated with environmental benefits of redispensing unused OADs. Environmental benefits were the largest when using a visual quality assurance procedure.

In the present study, 171 of 1071 patients (16.0%) returned unused OAD packages. This matches the literature on use patterns of patients using OADs. Approximately one-third of patients using OADs in a clinical setting discontinue their treatment early.^5,28,29^ Half these patients have redundant packages of OADs.^6^ Redispensing unused OAD packages thereby exceeds the waste-minimizing potential of other interventions, such as individualized dispensing strategies.^7^ The waste-minimizing potential could further increase if a larger proportion of patients would return medications, which depends on the type of medication and the return rate. For instance, postexposure prophylaxis therapy provided to medical students resulted in redispensing rates of approximately 74%,^30^ whereas redispensing rates of OADs were 2.6%. Focusing on such medications could thus increase the environmental benefits associated with redispensing. In addition, the return rate of unused packages could be increased, for instance, by personal reminders (eg, stickers, flyers, or an email) and logistical support (eg, pick-up service and disposal close to home).^11^ Nevertheless, the potential benefits must be balanced against the process costs and environmental outcomes, necessitating strategies to optimize these factors.

From an environmental perspective, it would be beneficial to minimize the process outcomes of quality assurance for redispensing.^9^ One approach would be to scale back on the quality measures taken (ie, seal bags and TTIs). However, successful implementation of redispensing unused OADs hinges significantly on stakeholder support, including patients and health care professionals who sought ensured drug quality for redispensing.^11,12,14^ Therefore, discussions with stakeholders are needed to establish appropriate quality measures based on convincing data. For instance, limiting temperature monitoring to temperature-sensitive medication was perceived as safe during the development of a clinical guideline,^25^ because of the absence of temperature breaches exceeding 30 °C in the trial and rare occurrences in other studies.^9,31^ However, this evidence is context specific and cannot be universally applied without further evidence due to climate variations. Moreover, donation programs in Greece and the US report positive outcomes based on merely visual quality checks,^26,27^ but a more comprehensive understanding of incidents (or lack thereof) is essential to implement redispensing of unused OADs based solely on visual checks. Another approach would be to seek alternative materials. This study showed that the environmental outcomes of the redispensing process were associated mainly with small electronic components (ie, resistors and capacitors) of TTIs; hence, using nonelectronic TTIs could be more environmentally beneficial. To illustrate, it is estimated that a nonelectronic TTI reduces environmental outcomes by 0.4% to 0.6% compared with electronic TTIs (eMethods in Supplement 2). In this way, the environmental benefits associated with redispensing unused OADs could be maximized. Implementing this form of TTI, however, depends on practical and safety factors in addition to the environmental outcome.

This study showed that redispensing unused OADs can be environmentally beneficial. However, extrapolation to other medications remains unclear because environmental outcomes of medications are hard to determine and few correlations have been identified to date. This study found large variability in environmental outcomes of OADs, caused primarily by variable API dosage per package and outcomes of API production. Production of 1 kg of API ranged from 57.6 of kg CO_2-eq_ for hydroxycarbamide to 1910.9 kg of CO_2-eq_ for temozolomide, which is in line with previous research on morphine and anesthetics.^32,33^ Redispensing was associated with relatively small environmental benefits in the optimized quality assurance procedure if the lowest API production outcomes from hydroxycarbamide were used as a proxy for all OADs. Hydroxycarbamide is one of the structurally simplest molecules and is produced using only a few steps. Most medications encompass more complex API molecules; hence, they will most likely have greater environmental outcomes and thus greater environmental benefits associated with redispensing than hydroxycarbamide. Accordingly, redispensing unused medications will presumably be beneficial for other medications as well, yet decisions on implementation should be based on the total benefit of redispensing, including cost benefits.^9^

### Strengths and Limitations

This study has some strengths. To our knowledge, this is the first study to assess the environmental outcome of waste-minimizing measures for medication, as well as the first to assess cradle-to-grave environmental outcomes of OADs. It could therefore support clinical decisions based on environmental outcomes. The systematic bottom-up LCA approach was particularly valuable to express the large variability of environmental outcomes of OADs because other environmental assessments (ie, input-output modeling) make distinctions only on the level of product groups. Another strength of this study is that data on the association of redispensing with waste (reduction) were retrieved from a multicenter trial encompassing a large patient cohort. Accordingly, this study provides insights into the outcomes of redispensing unused OADs as standard practice, providing both environmental and economic benefits.^9^

The study is also subject to limitations. Data on pharmaceutical production processes were lacking. As in previous studies,^32^ retrosynthesis and scale-up principles were followed to overcome this issue.^22^ Nevertheless, these strategies introduced large uncertainties, as assumptions were required. For instance, for each API, different patents, including availability of multiple retrosynthesis pathways with various starting materials, were found. Large variations among patents were found (eFigures 14 and 15 in Supplement 2), which was mitigated by using different extrapolation scenarios and applying sensitivity analyses (eFigures 16 and 17 in Supplement 2), showing similar trends of environmental outcomes of redispensing unused OADs. Because a high intervariability between environmental outcomes of OADs was found, the 1 of 2 inclusion rates in the trial may have caused a potential selection bias. This study corrected for this uncertainty by extrapolating the environmental outcomes of OADs per kilogram of active ingredient based on the minimum, mean, and maximum outcome, causing similar trends in the environmental benefits of redispensing unused OADs. Furthermore, pharmaceutical toxic effects in the environment associated with incorrect disposal routes of medication were not considered in this study, while OADs may potentially have ecotoxicologic effects.^16^ It could be argued that redispensing unused OADs promotes correct disposal routes because, in the redispensing program, specific patient instructions were provided to return unused OADs to the outpatient’s pharmacy. Consequently, redispensing may be associated with more environmental benefits than currently presented.

## Conclusions

In this quality improvement study, it was demonstrated that redispensing unused OADs could be associated with environmental benefits after process optimization. Accordingly, redispensing unused OADs could contribute to sustainability of cancer treatment through reduced costs and environmental outcomes. The redispensing program was currently tested for OADs, but it could also be environmentally beneficial for other medication groups; specifically, drugs that are frequently wasted or medications that have a high environmental burden of production could be interesting targets.

## Notes

Supplement 1. **eAppendix.** Inventory Data Calculations Sheet

Supplement 2. **eTable 1.** Oral Anticancer Drugs, Subclass and Storage Temperature Monitoring Included
eMethods.
**eFigure 1.** Redispensing Quality-Approved Oral Anticancer Drugs With Aid of a Time-Temperature Indicator, Sealed Packaging, and Patient Leaflet **eFigure 2.** Workflow to Calculate the Environmental Impact per OAD Package **eTable 2.** Dosage and Amount of Unit Doses per Package for the Six Modelled OADs **eFigure 3.** Modelled Reaction Pathway and Patents Used for Hydroxycarbamide **eFigure 4.** Modelled Reaction Pathway and Patents Used for Lenalidomide **eFigure 5.** Modelled Reaction Pathway and Patents Used for Olaparib **eFigure 6.** Modelled Reaction Pathway and Patents Used for Pomalidomide **eFigure 7.** Modelled Reaction Pathway and Patents Used for Sunitinib **eFigure 8.** Modelled Reaction Pathway and Patents Used for Temozolomide **eTable 3.** Average Data Used to Extrapolate to All OADs Used in the Trial for Damage to Human Health and Ecosystems and Climate Change **eFigure 9.** Relative Contribution Analysis of the Verification Materials on Damage to Human Health, Damage to Ecosystems and Climate Change per Dispensing in the Base Case Quality Assurance Procedure **eFigure 10.** Contribution Analysis for Tablet Production on (A) Damage to Human Health, (B) Damage to Ecosystems and (C) Climate Change for Six OADs **eFigure 11.** Contribution Analysis for API Production From Midpoint to Endpoint for (A) Damage to Human Health and (B) Damage to Ecosystems **eFigure 12.** Contribution Analysis for Tablet Production From Midpoint to Endpoint for (A) Damage to Human Health and (B) Damage to Ecosystems **eFigure 13.** Contribution Analysis for OAD Production From Midpoint to Endpoint for (A) Damage to Human Health and (B) Damage to Ecosystems **eFigure 14.** Two Modelled Reaction Pathways for Temozolomide, Including Used Patents **eFigure 15.** Results on API Production for Two Reaction Pathways for Temozolomide, Expressed per API Production on (A) Damage to Human Health, (B) Damage to Ecosystems and (C) Climate Change **eFigure 16.** Impacts per Patient per Year for the Trial Using API Production With Solvent Recovery for (A) Damage to Human Health, (B) Damage to Ecosystems and (C) Climate Change **eFigure 17.** Results on OAD Production Level Including Solvent Recovery, Expressed per OAD Package and on API Production Level Expressed per g API on (A) & (B) Damage to Human Health, (C) & (E) Damage to Ecosystems and (D) & (F) Climate Change
eReferences.

Supplement 3.
Data Sharing Statement
