# Supplementary material for: Environmental Outcomes of Reducing Medication Waste by Redispensing Unused Oral Anticancer Drugs
Source: JAMA Netw Open. 2024 Oct 10;7(10):e2438677. doi: 10.1001/jamanetworkopen.2024.38677 (PMC11581632; doi:10.1001/jamanetworkopen.2024.38677)
Supplement: Supplement 2. — eTable 1. Oral Anticancer Drugs, Subclass and Storage Temperature Monitoring Included eMethods. eFigure 1. Redispensing Quality-Approved Oral Anticancer Drugs With Aid of a Time-Temperature Indicator, Sealed Packaging, and Patient Leaflet eFigure 2. Workflow to Calculate the Environmental Impact per OAD Package eTable 2. Dosage and Amount of Unit Doses per Package for the Six Modelled OADs eFigure 3. Modelled Reaction Pathway and Patents Used for Hydroxycarbamide eFigure 4. Modelled Reaction Pathway and Patents Used for Lenalidomide eFigure 5. Modelled Reaction Pathway and Patents Used for Olaparib eFigure 6. Modelled Reaction Pathway and Patents Used for Pomalidomide eFigure 7. Modelled Reaction Pathway and Patents Used for Sunitinib eFigure 8. Modelled Reaction Pathway and Patents Used for Temozolomide eTable 3. Average Data Used to Extrapolate to All OADs Used in the Trial for Damage to Human Health and Ecosystems and Climate Change eFigure 9. Relative Contribution Analysis of the Verification Materials on Damage to Human Health, Damage to Ecosystems and Climate Change per Dispensing in the Base Case Quality Assurance Procedure eFigure 10. Contribution Analysis for Tablet Production on (A) Damage to Human Health, (B) Damage to Ecosystems and (C) Climate Change for Six OADs eFigure 11. Contribution Analysis for API Production From Midpoint to Endpoint for (A) Damage to Human Health and (B) Damage to Ecosystems eFigure 12. Contribution Analysis for Tablet Production From Midpoint to Endpoint for (A) Damage to Human Health and (B) Damage to Ecosystems eFigure 13. Contribution Analysis for OAD Production From Midpoint to Endpoint for (A) Damage to Human Health and (B) Damage to Ecosystems eFigure 14. Two Modelled Reaction Pathways for Temozolomide, Including Used Patents eFigure 15. Results on API Production for Two Reaction Pathways for Temozolomide, Expressed per API Production on (A) Damage to Human Health, (B) Damage to Ecosystems and (C) Climate Change eFigure 1 [file jamanetwopen-e2438677-s002.pdf]

Supplementary Online Content

Smale EM, Ottenbros AB, van den Bemt BJF, et al. Environmental outcomes of reducing medication waste by redispensing unused oral anticancer drugs. *JAMA Netw Open.* 2024;7(10):e2438677. doi:10.1001/jamanetworkopen.2024.38677

eTable 1. Oral Anticancer Drugs, Subclass and Storage Temperature Monitoring Included
eMethods.
eFigure 1. Redispensing Quality-Approved Oral Anticancer Drugs With Aid of a Time-Temperature Indicator, Sealed Packaging, and Patient Leaflet
eFigure 2. Workflow to Calculate the Environmental Impact per OAD Package
eTable 2. Dosage and Amount of Unit Doses per Package for the Six Modelled OADs
eFigure 3. Modelled Reaction Pathway and Patents Used for Hydroxycarbamide
eFigure 4. Modelled Reaction Pathway and Patents Used for Lenalidomide
eFigure 5. Modelled Reaction Pathway and Patents Used for Olaparib
eFigure 6. Modelled Reaction Pathway and Patents Used for Pomalidomide
eFigure 7. Modelled Reaction Pathway and Patents Used for Sunitinib
eFigure 8. Modelled Reaction Pathway and Patents Used for Temozolomide
eTable 3. Average Data Used to Extrapolate to All OADs Used in the Trial for Damage to Human Health and Ecosystems and Climate Change
eFigure 9. Relative Contribution Analysis of the Verification Materials on Damage to Human Health, Damage to Ecosystems and Climate Change per Dispensing in the Base Case Quality Assurance Procedure.
eFigure 10. Contribution Analysis for Tablet Production on (A) Damage to Human Health, (B) Damage to Ecosystems and (C) Climate Change for Six OADs
eFigure 11. Contribution Analysis for API Production From Midpoint to Endpoint for (A) Damage to Human Health and (B) Damage to Ecosystems
eFigure 12. Contribution Analysis for Tablet Production From Midpoint to Endpoint for (A) Damage to Human Health and (B) Damage to Ecosystems
eFigure 13. Contribution Analysis for OAD Production From Midpoint to Endpoint for (A) Damage to Human Health and (B) Damage to Ecosystems
eFigure 14. Two Modelled Reaction Pathways for Temozolomide, Including Used Patents

eFigure 15. Results on API Production for Two Reaction Pathways for Temozolomide, Expressed per API Production on (A) Damage to Human Health, (B) Damage to Ecosystems and (C) Climate Change

eFigure 16. Impacts per Patient per Year for the Trial Using API Production With Solvent Recovery for (A) Damage to Human Health, (B) Damage to Ecosystems and (C) Climate Change

eFigure 17. Results on OAD Production Level Including Solvent Recovery, Expressed per OAD Package and on API Production Level Expressed per g API on (A) & (B) Damage to Human Health, (C) & (E) Damage to Ecosystems and (D) & (F) Climate Change

© 2024 Smale EM et al. *JAMA Network Open.*

eReferences.

This supplementary material has been provided by the authors to give readers additional information about their work.

© 2024 Smale EM et al. *JAMA Network Open.*

eTable 1. Oral Anticancer Drugs, Subclass and Storage Temperature Monitoring Included. Table is extracted from Smale et al.[1].

Generic name	Branded name	ATC	Subclass	Storage temperature monitoring (ºC)[a]
Abemaciclib	Verzenios	L01EF03	Targeted therapies	8 – 40
Abirateron	Zytiga	L02BX03	Endocrine therapy	8 – 40
Afatinib	Giotrif	L01EB03	Targeted therapies	8 – 40
Alectinib	Alecensa	L01ED03	Targeted therapies	8 – 40
Anagrelide	Xagrid	L01XX35	Cytotoxic agents	8 – 40
Anastrozol	Anastrozol	L02BG03	Endocrine therapy	8 – 30
Apalutamide	Erleada	L02BB05	Endocrine therapy	8 – 40
Axitinib	Inlyta	L01EK01	Targeted therapies	8 – 40
Binimetinib	Mektovi	L01EE03	Targeted therapies	8 – 40
Bosutinib	Bosulif	L01EA04	Targeted therapies	8 – 40
Cabozantinib	Cabometyx	L01EX07	Targeted therapies	8 – 25
Crizotinib	Xalkori	L01ED01	Targeted therapies	8 – 40
Cyclosfosfamide	Endoxan	L01AA01	Cytotoxic agents	8 – 25
Dabrafenib	Tafinlar	L01EC02	Targeted therapies	8 – 40
Dasatinib	Sprycel	L01EA02	Targeted therapies	8 – 40
Exemestaan		L02BG06	Endocrine therapy	8 – 30
Encorafenib	Braftovi	L01EC03	Targeted therapies	8 – 30
Enzalutamide	Xtandi	L02BB04	Endocrine therapy	8 – 40
Erlotinib		L01EB02	Targeted therapies	8 – 25
Everolimus	Afinitor	L01EG02	Targeted therapies	8 – 30
Gefitinib	Iressa	L01EB01	Targeted therapies	8 – 30
Hydroxycarbamide	Hydroxyurea, Hydrea, Siklos	L01XX05	Cytotoxic agents	8 – 30
Ibrutinib	Imbruvica	L01EL01	Targeted therapies	8 – 40
Imatinib	Glivec	L01EA01	Targeted therapies	8 – 25
Ixazomib	Ninlaro	L01XG03	Targeted therapies	8 – 25
Lenalidomide	Revlimid	L04AX04	Immunosuppressants	8 – 40
Lenvatinib	Kisplyx, Lenvima	L01EX08	Targeted therapies	8 – 25
Letrozol		L02BG04	Endocrine therapy	8 – 40
Lorlatinib	Lorviqua	L01ED05	Targeted therapies	8 – 40
Midostaurine	Rydapt	L01EX10	Targeted therapies	8 – 40
Mitotaan	Lysodren	L01XX23	Cytotoxic agents	8 – 40
Nilotinib	Tasigna	L01EA03	Targeted therapies	8 – 30
Niraparib	Zejula	L01XK02	Targeted therapies	8 – 30
Olaparib	Lynparza	L01XK01	Targeted therapies	8 – 40*
Osimertinib	Tagrisso	L01EB04	Targeted therapies	8 – 40
Palbociclib	Ibrance	L01EF01	Targeted therapies	8 – 40

© 2024 Smale EM et al. *JAMA Network Open.*

Generic name	Branded name	ATC	Subclass	Storage temperature monitoring (ºC) [a]
Pazopanib	Votrient	L01EX03	Targeted therapies	8 – 40
Pomalidomide	Imnovid	L04AX06	Immunosuppressants	8 – 40
Ponatinib	Iclusig	L01EA05	Targeted therapies	8 – 40
Procarbazine	Natulan	L01XB01	Cytotoxic agents	8 – 25
Regorafenib	Stivarga	L01EX05	Targeted therapies	8 – 40
Ruxolitinib	Jakavi	L01EJ01	Targeted therapies	8 – 30
Sorafenib	Nexavar	L01EX02	Targeted therapies	8 – 25
Sunitinib	Sutent	L01EX01	Targeted therapies	8 – 40
Tamoxifen		L02BA01	Endocrine therapy	8 – 25
Tegafur/gimeracil/oteracil	Teysuno	L01BC53	Cytotoxic agents	8 – 40
Temozolomide		L01AX03	Cytotoxic agents	8 – 30
Trifluridine/tipiracil	Lonsurf	L01BC59	Cytotoxic agents	8 – 40
Vandetanib	Caprelsa	L01EX04	Targeted therapies	8 – 30
Venetoclax	Venclyxto	L01XX52	Targeted therapies	8 – 40
Vismodegib	Erivedge	L01XJ01	Targeted therapies	8 – 25

[a] Storage temperature monitoring was based on storage requirements stated in the Summary of Product Characteristics. If no maximum temperature was described in the SmPC, a maximum storage temperature of 40ºC was used, corresponding to stability research (European Drugs Agency ICH Q1A(R2) Guideline 2003).

[b] only Olaparib tablets were included (e.g. not capsules)

© 2024 Smale EM et al. *JAMA Network Open.*

eMethods.

Quality Assurance Procedures

Quality of oral anticancer drugs (OADs) upon storage at home can be affected by various factors, including humidity, light, introduction of counterfeits and storage temperature breaches. One main and two additional quality scenarios were analysed in this study to account for these factors, ensuring that only good-quality medication was redispensed. In all scenarios, medication was protected from influences of humidity and light by the original manufacturing package. Furthermore, authenticity of drugs was assured by the seal of the original medication package and/or the intactness of the additional sealed outer packaging (Transposafe, Bracy Co) shown in eFigure 1. Finally, storage temperature was monitored in the base case quality assurance procedure as well as in the optimized quality assurance procedure with aid of a time-temperature indicator (TTI) (Libero ITS [customer configuration], Elpro-Buchs AG), shown in eFigure 1. Each dispensing was accompanied with a patient leaflet, explaining how to store the medication appropriately and how to return medication in case it is remaining unused (eFigure 1). Patients were asked to return unused drugs during their next hospital visit. Therefore, it was assumed that no additional transport or at home disposal took place and were excluded from assessment.

© 2024 Smale EM et al. *JAMA Network Open.*

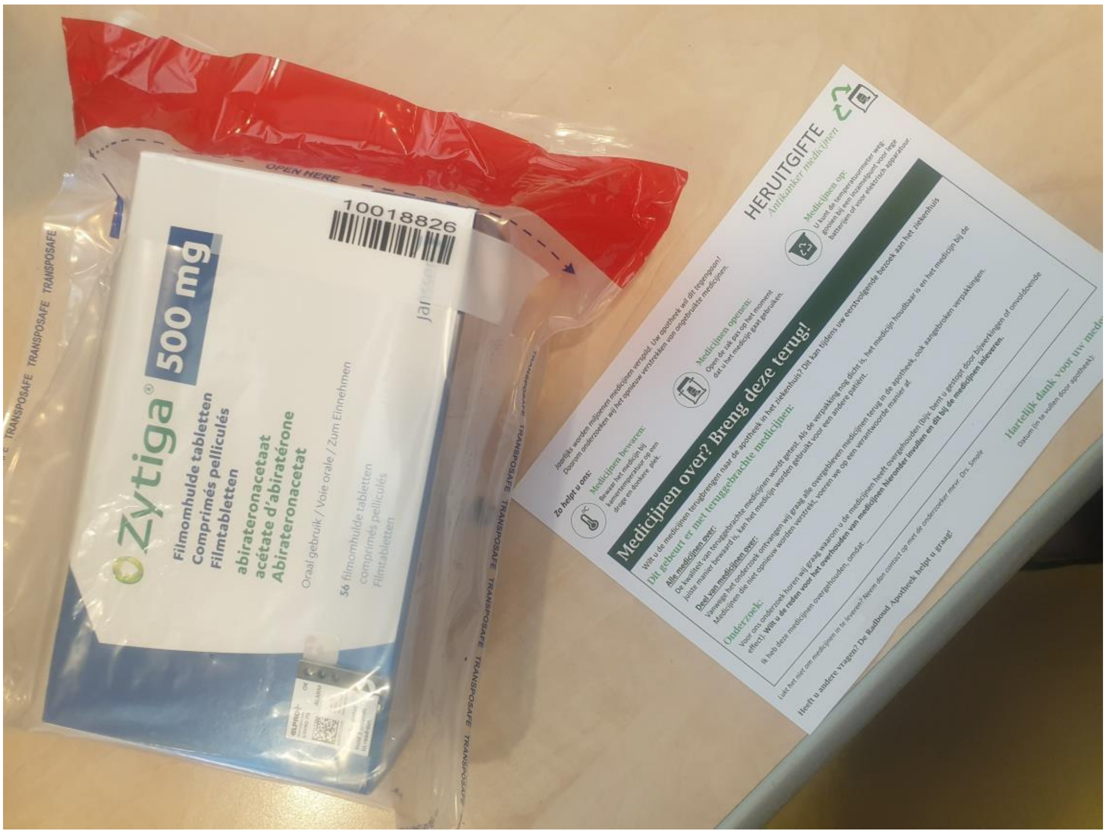

eFigure 1. Redispensing Quality-Approved Oral Anticancer Drugs With Aid of a Time-Temperature Indicator, Sealed Packaging, and Patient Leaflet

Collecting inventory data for life cycle assessment

Quality assurance materials

Data on the time-temperature indicator was gathered from separating and weighting the separated pieces. The manufacturer provided information on production sites and transport routes, but this data was confidential and will therefore not be described in more detail. The seal bag is made from low density polyethylene and assumed to be produced in Poland. The flyer is printed locally at the pharmacy. Specific inventory details can be found in 'Inventory Data Calculation' sheet[2].

Oral anticancer drugs

Due to data collection being a labor-intensive procedure, data from six active pharmaceutical ingredients (APIs) was used to extrapolate to all the OADs used in the trial (eTable 1). The environmental impact, expressed in damage to human health, damage to ecosystems and climate change was calculated with ReCiPe 2016 (H)[3]. By multiplying environmental impacts per kg API with the dosage (i.e., mg API per unit dose), the impact per OAD unit dose was obtained. Finally, this number was multiplied with the number of unit doses (e.g., tablets or capsules) per OAD package (eTable 2). To obtain total environmental impacts per OAD package, impacts of the blister and carton medication box were added (eFigure 2). As the weight and number of blisters per OAD package varied, the mean environmental impact for these variables was used in the extrapolation from six OADs to all OADs used in the trial (eTable 3). Finally, international transport to the Netherlands was considered, as well as national transport from the wholesaler to the pharmacy. Disposal of OADs and other waste was assumed to take place in the Netherlands through the pharmacy. Blisters containing tablets, as well as the disposal bins in which unused drugs were collected, were assumed to be incinerated as chemical hazardous waste. Incineration of OAD packages that were disapproved of quality would have occurred in standard practice as well and were therefore excluded in the analysis.

Life cycle inventory: API production

To build the life cycle inventory of the six selected APIs, a similar approach of Parvatker et al[4] was used. First, patent data found via Reaxys[5] gave the resources needed for the production of the APIs. Background data for each resource was taken from EcoInvent v3.8[6,7], specifically system model "allocation at point of substitution" was used. If chemicals used in the patents were not present in the EcoInvent dataset, new patents were searched to identify its production pathway. Patents were selected based on production feasibility and patent publication year. The selected production pathways were checked on feasibility on industrial scale by a chemical expert. eFigure 3 to eFigure 8 show the reaction pathways and different patents used to model the six APIs. It must be noted that actual industrial production processes remain unsure without company specific information. Therefore, this study does not claim that the used patents comprise the only production pathway possible and used scenario and sensitivity analyses to account for this uncertainty. For example, hydroxycarbamide could potentially also be made directly from urea with hydroxylamine. However, no patent was found to support this reaction route and hence the reaction pathway of eFigure 3 was used.

Second, inventory data was predicted for up-scaled production (1000L reactor) with the framework of Piccinno et al.[8]. General assumptions were made to scale up lab protocols to industrial scale for the selected patents. The yield was taken from the patent, masses of reagents were linearly scaled and solvents were reduced by 20% on industrial scale[8]. In some cases, a larger solvent reduction was assumed, as unrealistic amounts of solvents were needed to produce 1 kg API (e.g., >500 kg solvent per kg API). Solvent recycling was not considered as this can only be done to a limited extend in pharmaceutical manufacturing due to the risk of introducing impurities[9]. Energy requirements were based on the description in the patent and formulas described in Piccinno et al.[8]. The heat capacity value was taken for the most prominent solvent. In case no production country was known, default distances were selected based on the International Standard of Industrial Classification (ISIC) groups[10] and general transport distances mentioned by the Ecoinvent V3 transport report[11]. In general, it was assumed that APIs are produced in India[12]. Transport in modelling the reagents is not included, as it was assumed to take place in a multi-step process. The specific assumptions and calculations can be found in LCI data [2].

Life cycle inventory: galenic formulation

The type of excipients for galenic formulation were taken from the Summary of Product Characteristics of each OAD and the amounts were based on Wang et al[13]. In general, galenic formulation was assumed to take place in India[12]. For the filling and outer shell production the production of gelatine and mannitol background processes were modelled separately. The production of the gelatine was modelled based on literature data[14]. For mannitol, LCA data of sorbitol production was used as a proxy[15]. For excipients that were not present in the EcoInvent V3.8

background process database, proxy processes were used. Specific calculation and modelling details can be found in LCI data [2].

Sensitivity analysis solvent recovery

In the LCA, solvent recycling was not taken into account as this can only be done to a limited extend in pharmaceutical manufacturing due to the risk of introducing impurities[9]. In chemical reactions, however, solvent recycling is common, with an average recovery rate of 68%[8]. A scenario including solvent recovery in the API production processes was explored. Effects of these variations on damage to human health, damage to ecosystems and climate change are displayed in eFigure6 and eFigure7.

Sensitivity analysis non-electronic TTI

Inventory data for a non-electronic time-temperature indicator was gathered and calculations are provided in the 'Inventory Data Calculation' sheet[2].

© 2024 Smale EM et al. *JAMA Network Open.*

eFigure 2. Workflow to Calculate the Environmental Impact per OAD Package

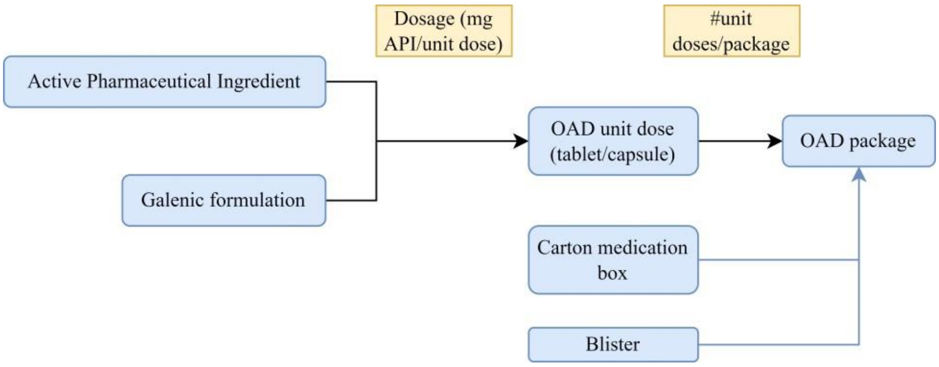

© 2024 Smale EM et al. *JAMA Network Open.*

eTable 2. Dosage and Amount of Unit Doses per Package for the Six Modelled OADs

OAD	Dosage (mg API per unit dose)	# unit doses per package
Hydroxycarbamide	500	100
Lenalidomide	10	21
Olaparib	150	56
Pomalidomide	4	21
Sunitinib	12.5	28
Temozolomide	100	5

© 2024 Smale EM et al. *JAMA Network Open.*

eFigure 3. Modelled Reaction Pathway and Patents Used for Hydroxycarbamide

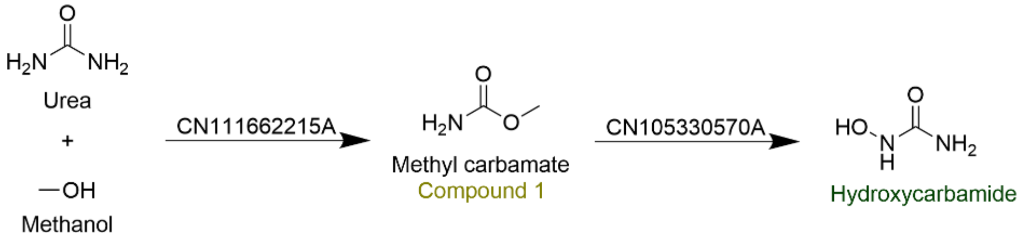

© 2024 Smale EM et al. *JAMA Network Open.*

eFigure 4. Modelled Reaction Pathway and Patents Used for Lenalidomide

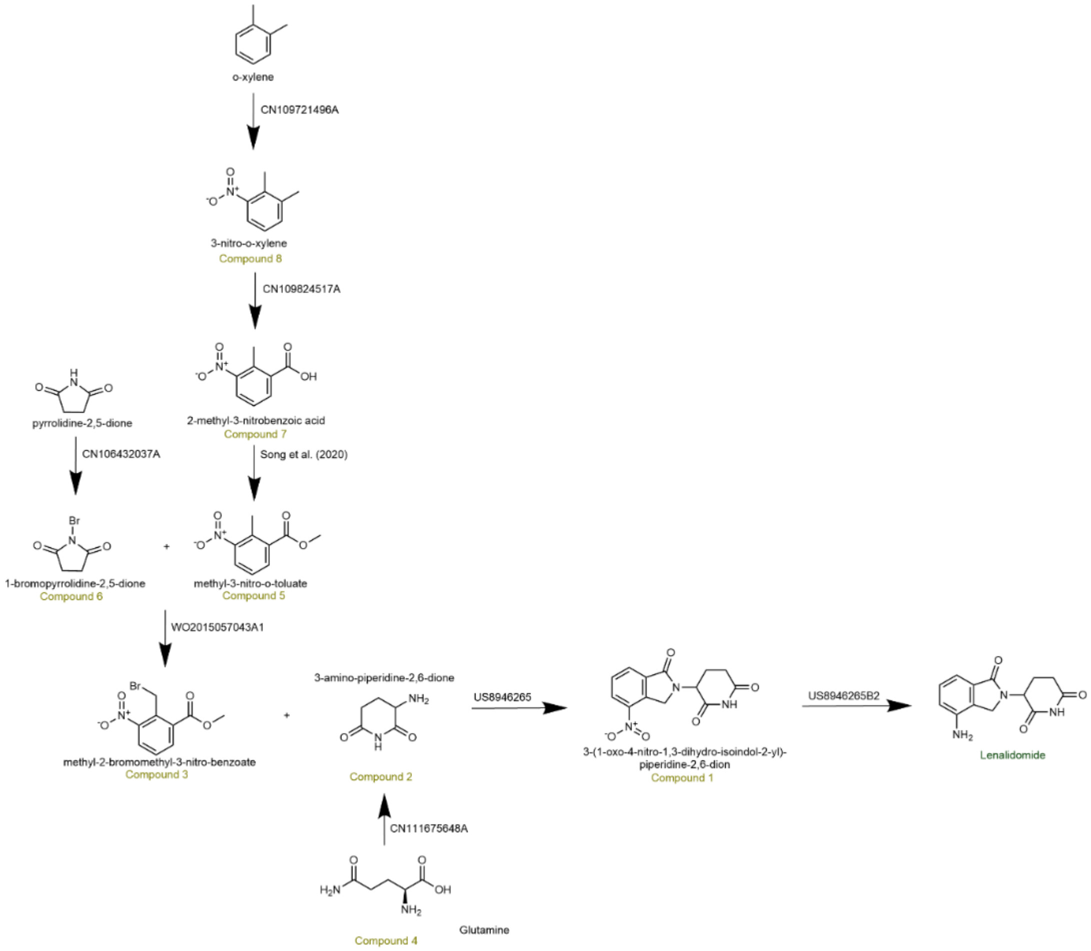

© 2024 Smale EM et al. *JAMA Network Open.*

eFigure 5. Modelled Reaction Pathway and Patents Used for Olaparib

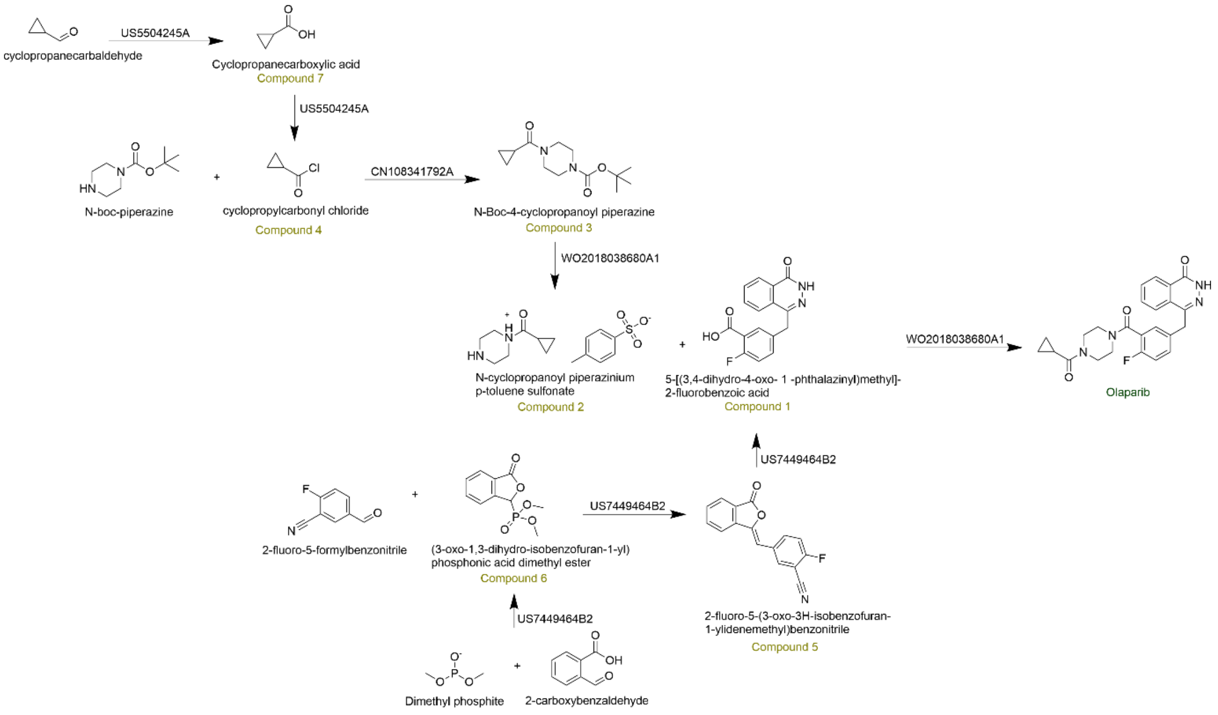

© 2024 Smale EM et al. *JAMA Network Open.*

eFigure 6. Modelled Reaction Pathway and Patents Used for Pomalidomide

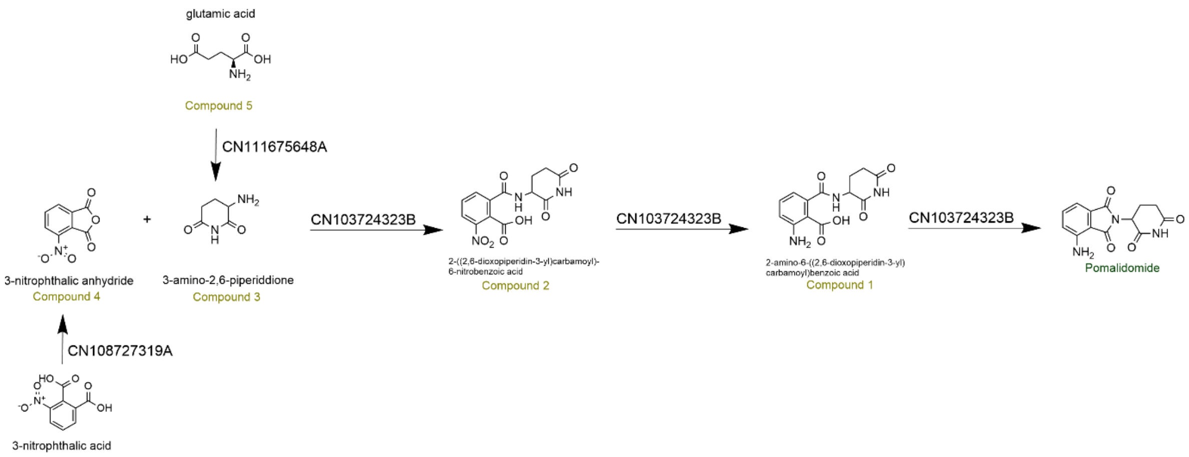

© 2024 Smale EM et al. *JAMA Network Open.*

eFigure 7. Modelled Reaction Pathway and Patents Used for Sunitinib

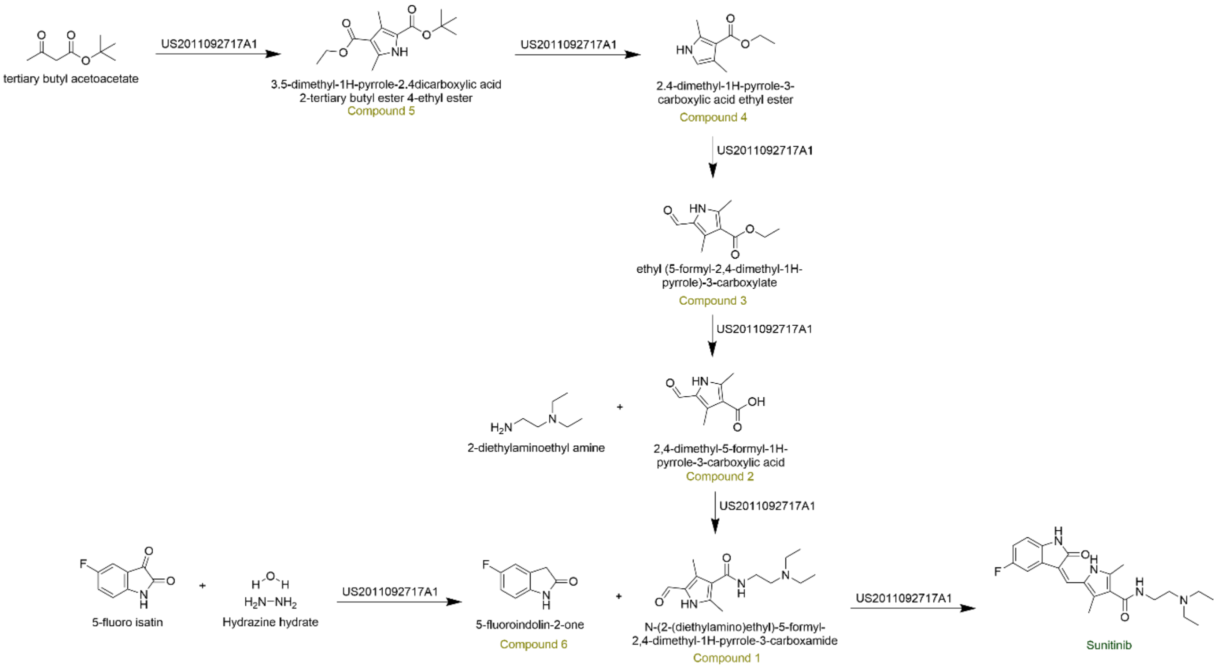

© 2024 Smale EM et al. *JAMA Network Open.*

eFigure 8. Modelled Reaction Pathway and Patents Used for Temozolomide

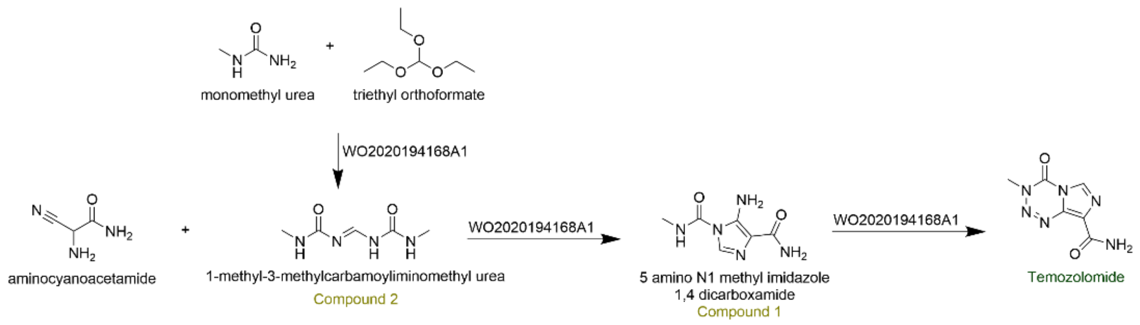

© 2024 Smale EM et al. *JAMA Network Open.*

eTable 3. Average Data Used to Extrapolate to All OADs Used in the Trial for Damage to Human Health and Ecosystems and Climate Change

	Blister [a]	Galenic formulation	Medicine box	Incineration	Patient leaflet	TTI	Seal bag	Lowest API	Mean API	Highest API
Unit	Per piece	Per kg API	Per piece	Per piece	Per piece	Per piece	Per piece	Per kg API	Per kg API	Per kg API
kg $CO_{2\text{-}eq}$	2.67E-02	2.38E+02	2.75E-02	2.45E-01	1.79E-02	6.88E-01	2.86E-02	5.76E+01	7.65E+02	1.91E+03
DALY	7.52E-08	6.13E-04	1.10E-07	4.27E-07	4.72E-08	2.40E-06	7.39E-08	1.40E-04	1.56E-03	3.75E-03
species.year	1.22E-10	1.34E-06	2.58E-10	9.02E-10	1.04E-10	3.45E-09	1.38E-10	2.56E-07	2.74E-06	7.81E-06

[a] The average amount of blisters (6 strips) is taken for all OADs.

© 2024 Smale EM et al. *JAMA Network Open.*

eFigure 9. Relative Contribution Analysis of the Verification Materials on Damage to Human Health, Damage to Ecosystems and Climate Change per Dispensing in the Base Case Quality Assurance Procedure. The Main Contributor to All Impact Categories Is the Time-Temperature Indicator, due to the Use of Electric Components.

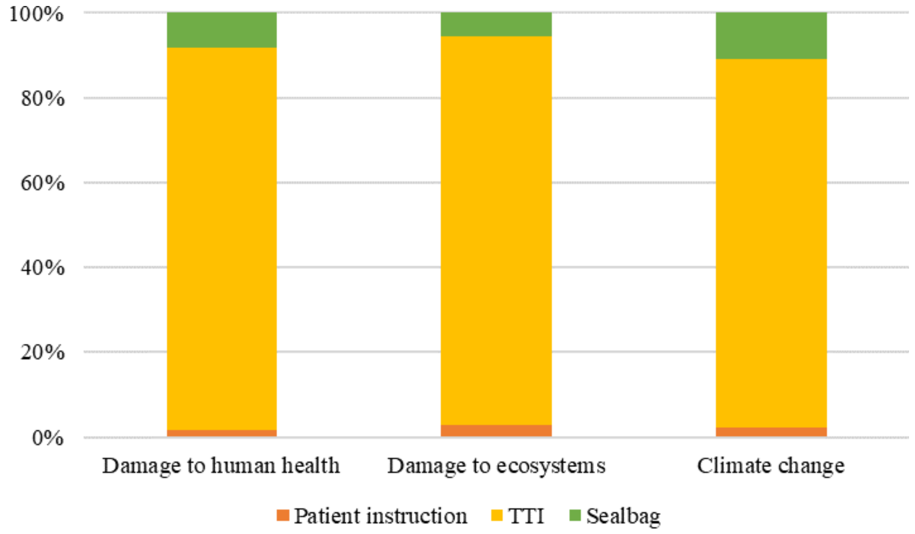

© 2024 Smale EM et al. *JAMA Network Open.*

eFigure 10. Contribution Analysis for Tablet Production on (A) Damage to Human Health, (B) Damage to Ecosystems and (C) Climate Change for Six OADs. The Environmental Impact per Tablet Results From the Impact of API Production per Dosage Combined With the Impact of Galenic Formulation.

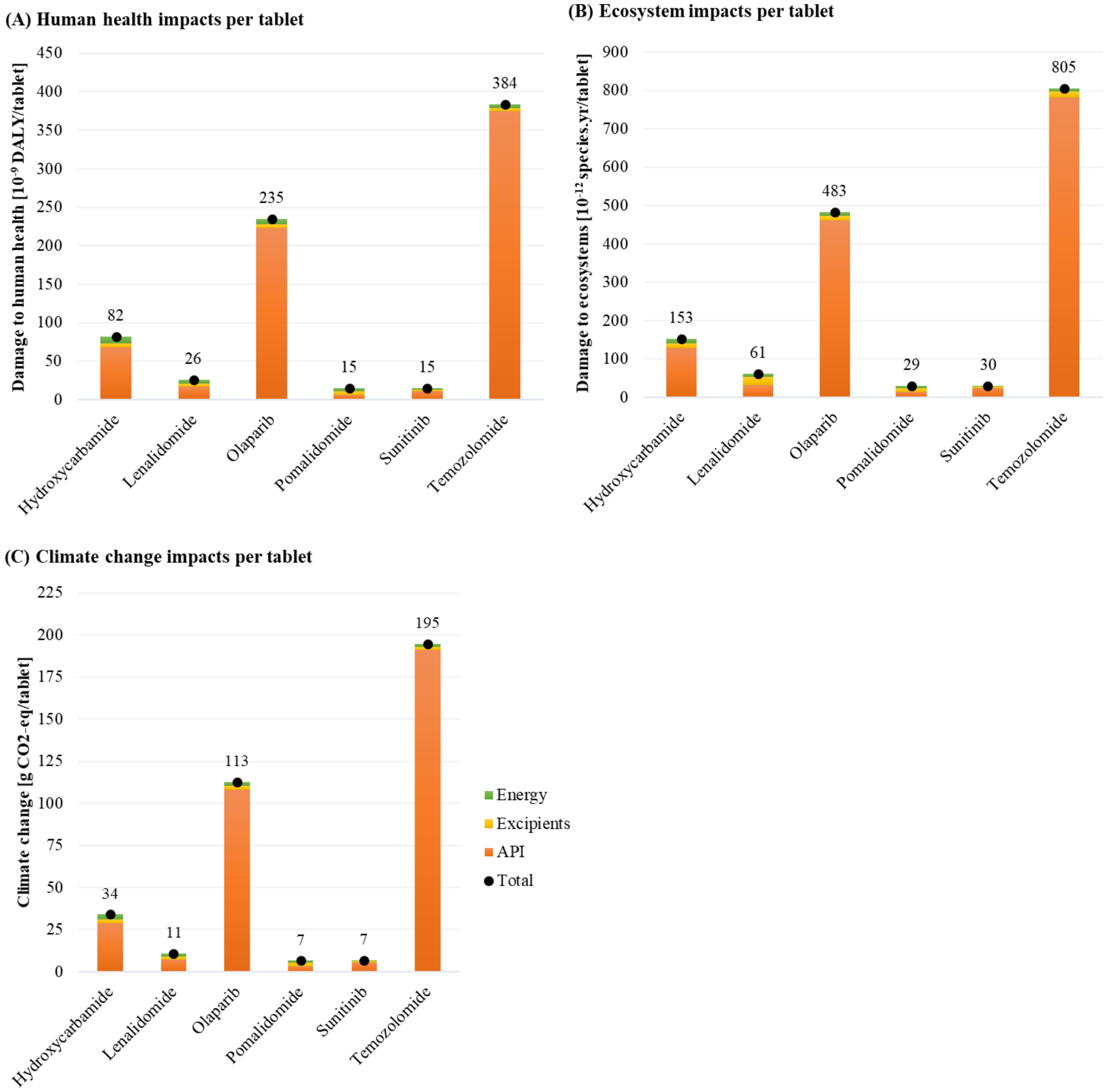

© 2024 Smale EM et al. *JAMA Network Open.*

eFigure 11. Contribution Analysis for API Production From Midpoint to Endpoint for (A) Damage to Human Health and (B) Damage to Ecosystems. From this Contribution Analyses, it Was Determined That Climate Change Contributed Most to Both Damage to Human Health and Ecosystems and Could Most Conveniently Be Used to Compare Results With Other Studies. Hence, Climate Change Was Selected to Elaborate on in the Main Article.

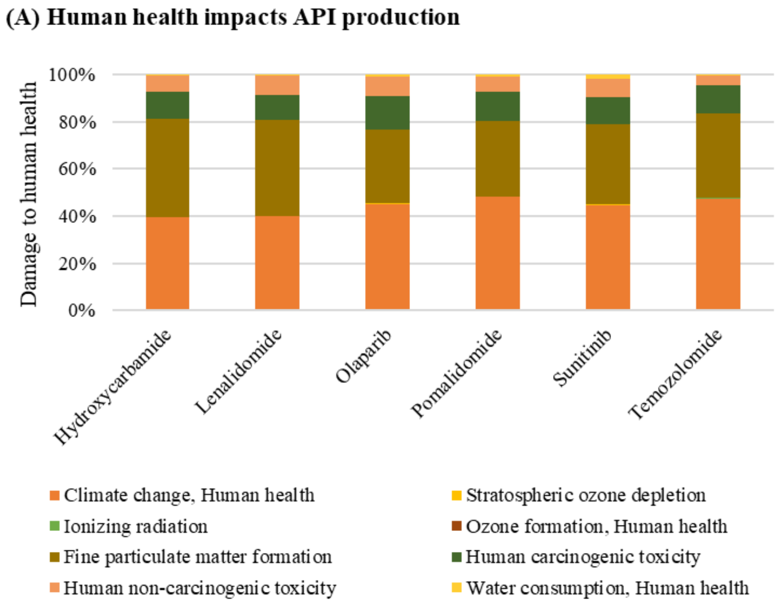

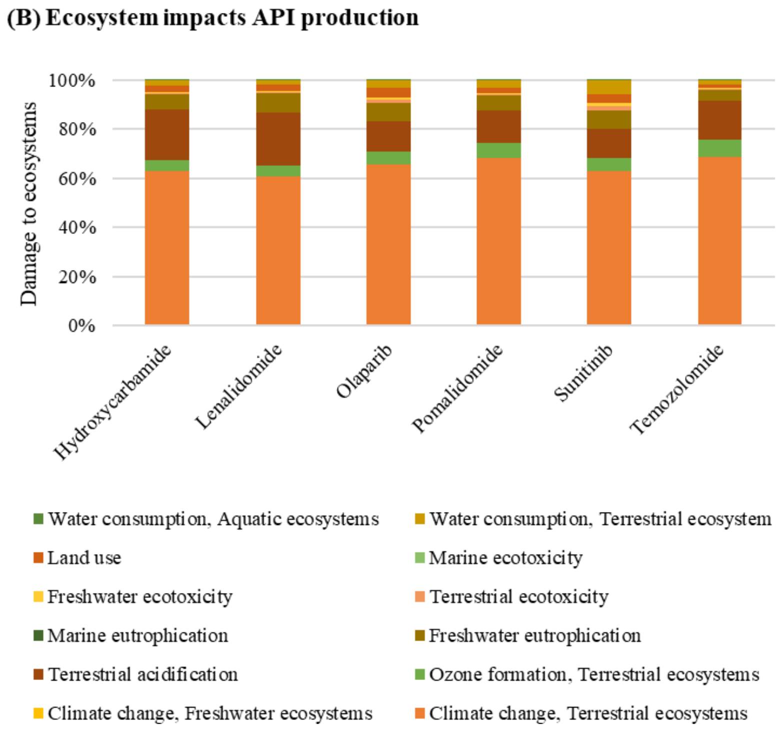

© 2024 Smale EM et al. *JAMA Network Open.*

eFigure 12. Contribution Analysis for Tablet Production From Midpoint to Endpoint for (A) Damage to Human Health and (B) Damage to Ecosystems. From This Contribution Analyses, it Was Determined That Climate Change Contributed Most to Both Damage to Human Health and Ecosystems and Could Most Conveniently Be Used to Compare Results With Other Studies. Hence, Climate Change Was Selected to Elaborate on in the Main Article.

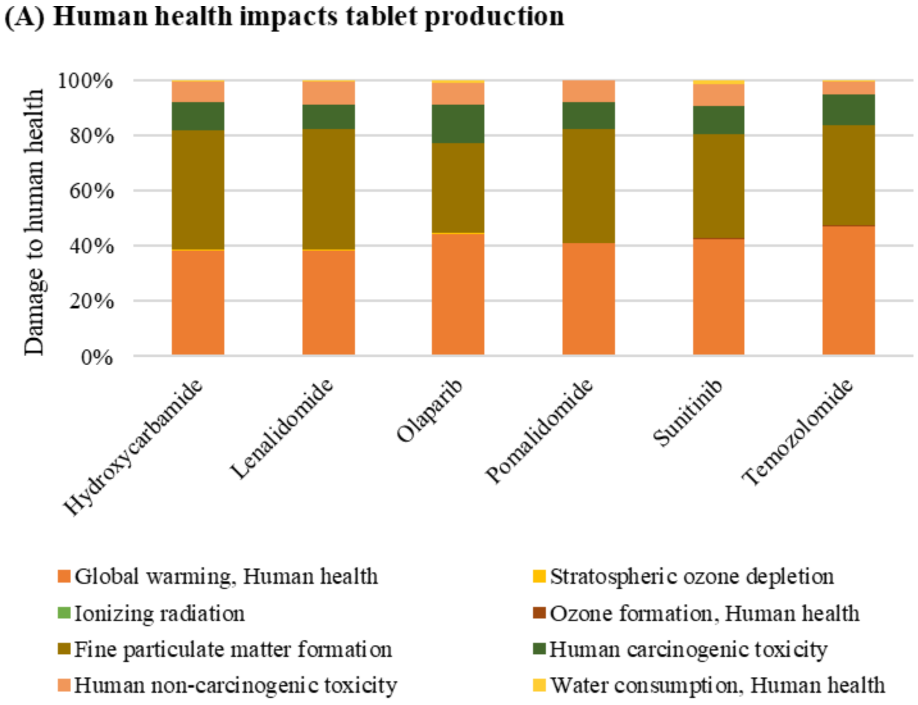

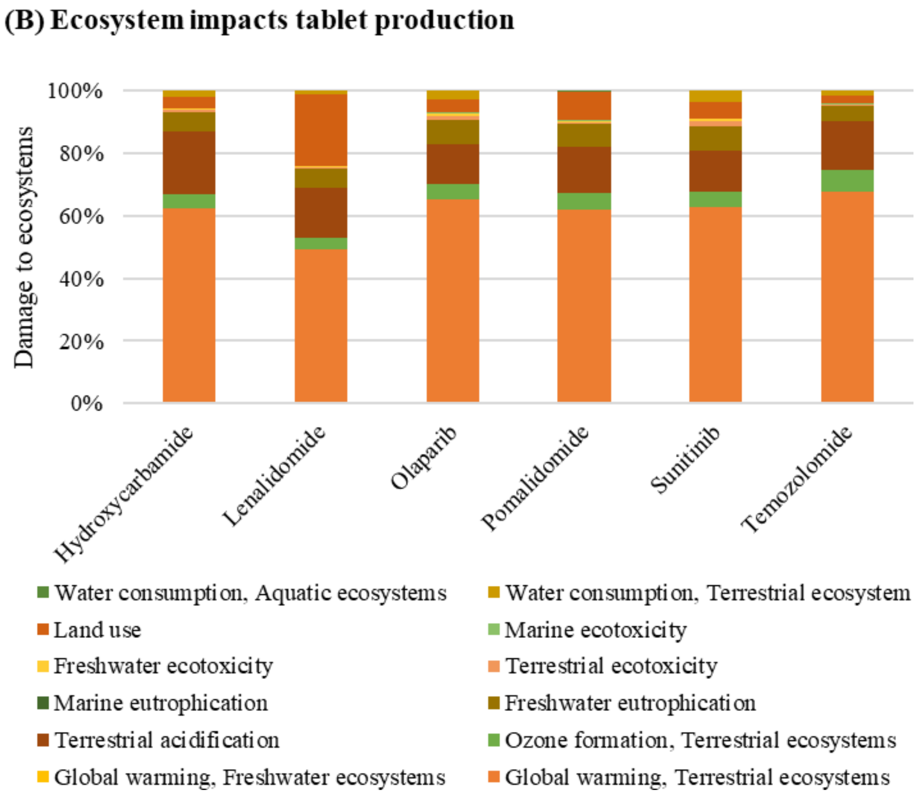

© 2024 Smale EM et al. *JAMA Network Open.*

eFigure 13. Contribution Analysis for OAD Production From Midpoint to Endpoint for (A) Damage to Human Health and (B) Damage to Ecosystems. From This Contribution Analyses, it Was Determined That Climate Change Contributed Most to Both Damage to Human Health and Ecosystems and Could Most Conveniently Be Used to Compare Results With Other Studies. Hence, Climate Change Was Selected to Elaborate on in the Main Article.

(A) Human health impacts OAD production

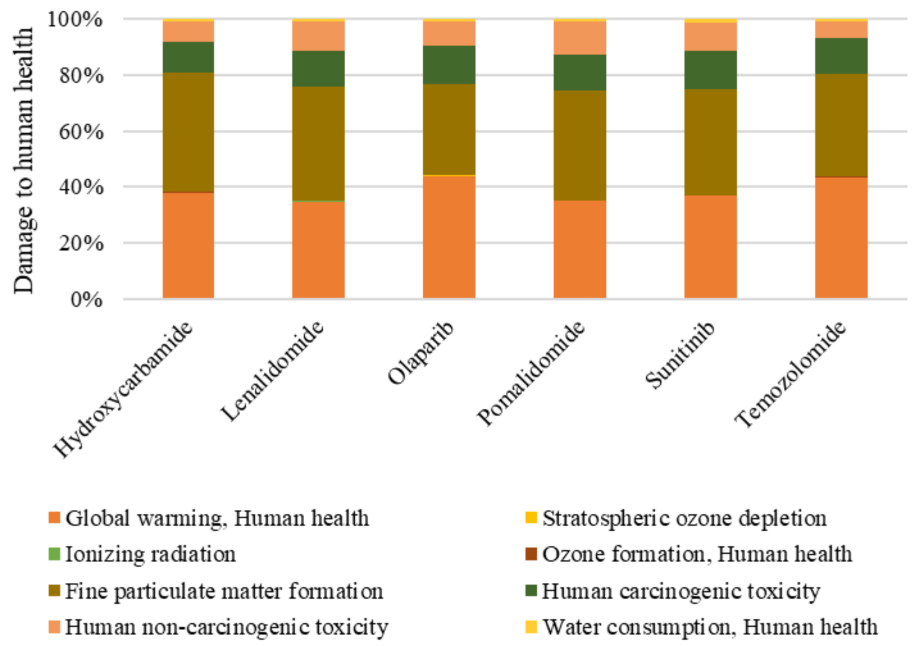

(B) Ecosystem impacts OAD production

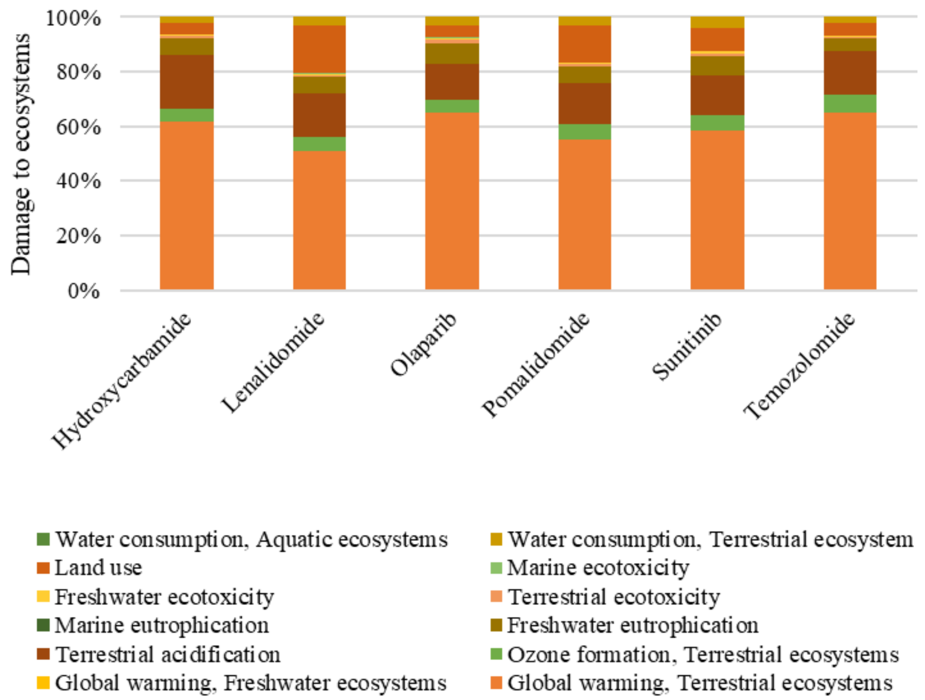

© 2024 Smale EM et al. *JAMA Network Open.*

eFigure 14. Two Modelled Reaction Pathways for Temozolomide, Including Used Patents. To Verify the Effect of Using a Different Patent for Determining the Environmental Impacts of an OAD, a Test-Case With Temozolomide Was Performed. The Reason for Checking This Was That Two Suitable Patents for Producing Temozolomide Appeared.

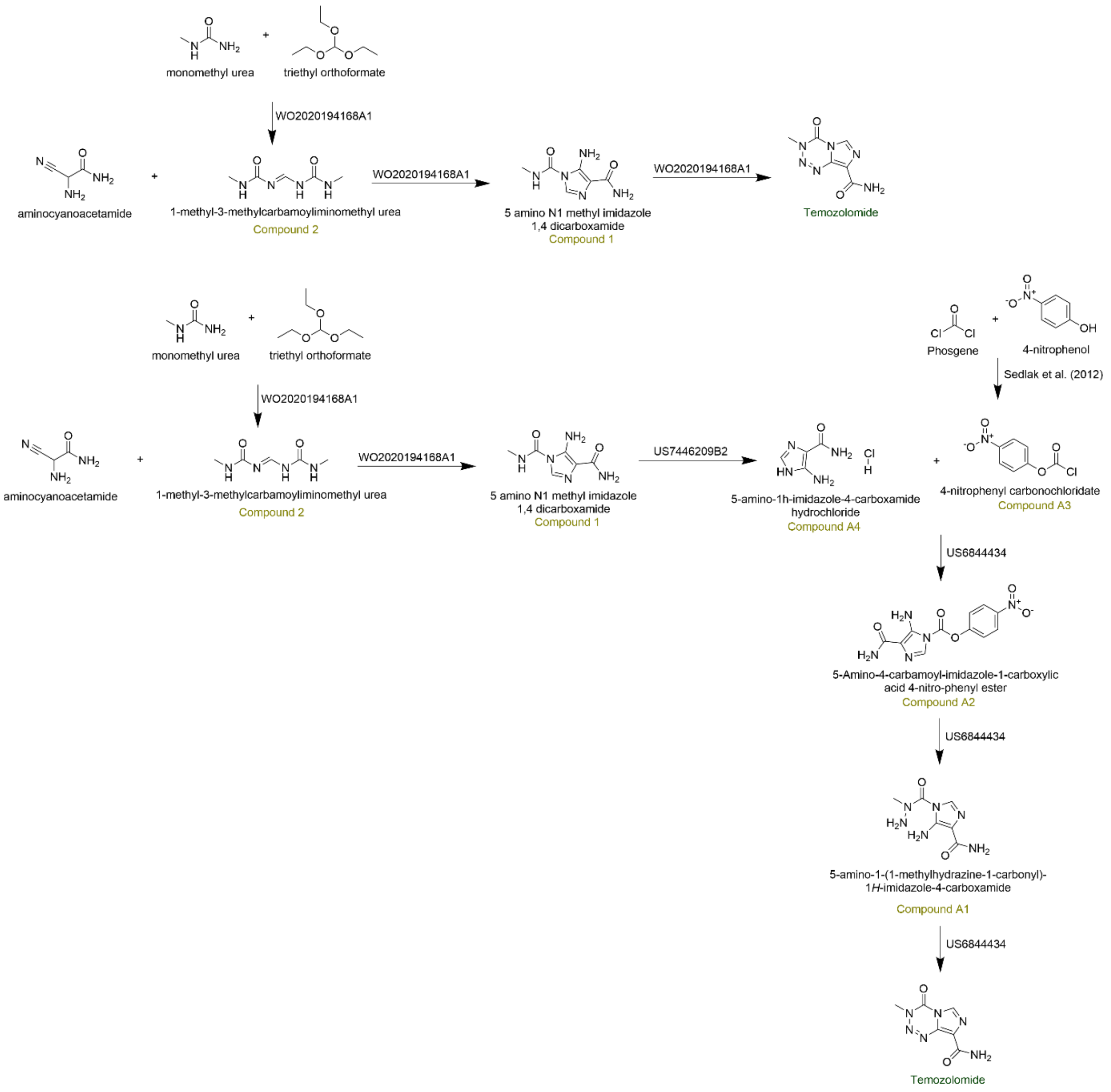

© 2024 Smale EM et al. *JAMA Network Open.*

eFigure 15. Results on API Production for Two Reaction Pathways for Temozolomide, Expressed per API Production on (A) Damage to Human Health, (B) Damage to Ecosystems and (C) Climate Change. The Alternative Chemical Reaction Provided Considerably Higher Environmental Impacts, as Shown in eFigure 16. This Mainly Related to a Large Amount of Organic Solvent Used in the Alternative Production Pathway. Given the Profit-Driven Structure of the Pharmaceutical Industry, Using Such Suboptimal Amounts of Organic Solvents Was Perceived Unlikely, Therefore the Outcomes of the Original Route Were Used. However, This Example Does Show That the LCA Method Is Prone to Specific Assumptions That May Have Introduced a Bias in the Outcomes.

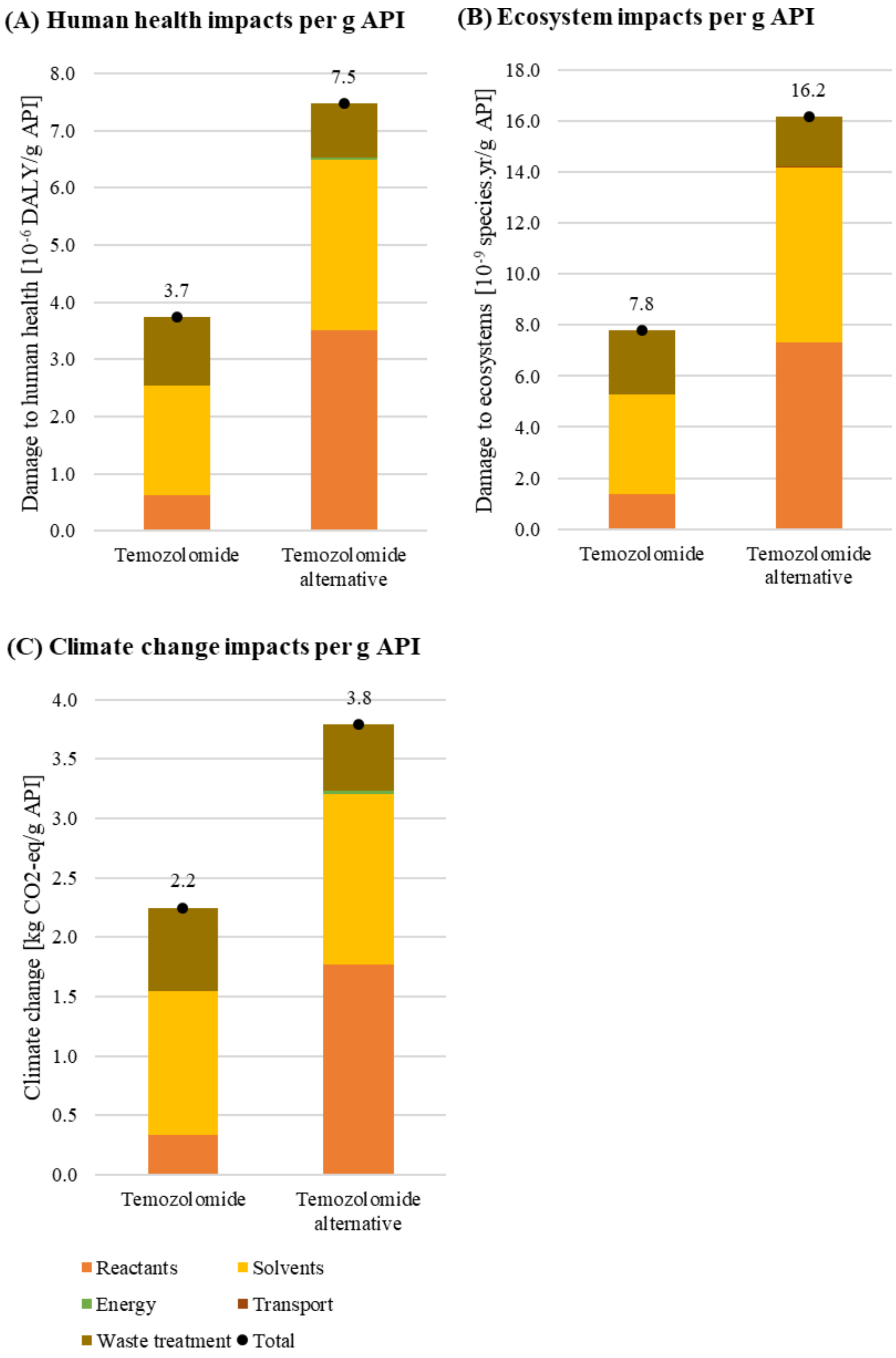

© 2024 Smale EM et al. *JAMA Network Open.*

eFigure 16. Impacts per Patient per Year for the Trial Using API Production With Solvent Recovery for (A) Damage to Human Health, (B) Damage to Ecosystems and (C) Climate Change. Regardless of Recovering Solvents During API Production, Similar Environmental Impacts Were Shown for All Three Quality Assurance Procedures. In Other Words, the Uncertainty of Solvents Recovery Did Not Affect the Results Displayed by This Study.

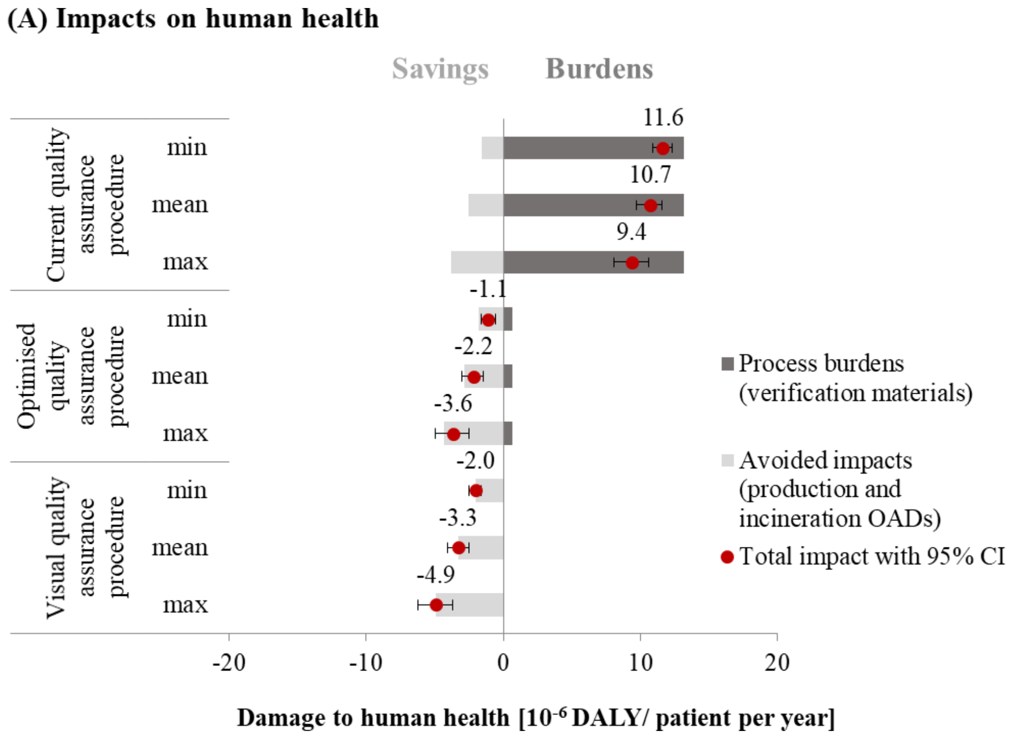

© 2024 Smale EM et al. *JAMA Network Open.*

(B) Impacts on ecosystems

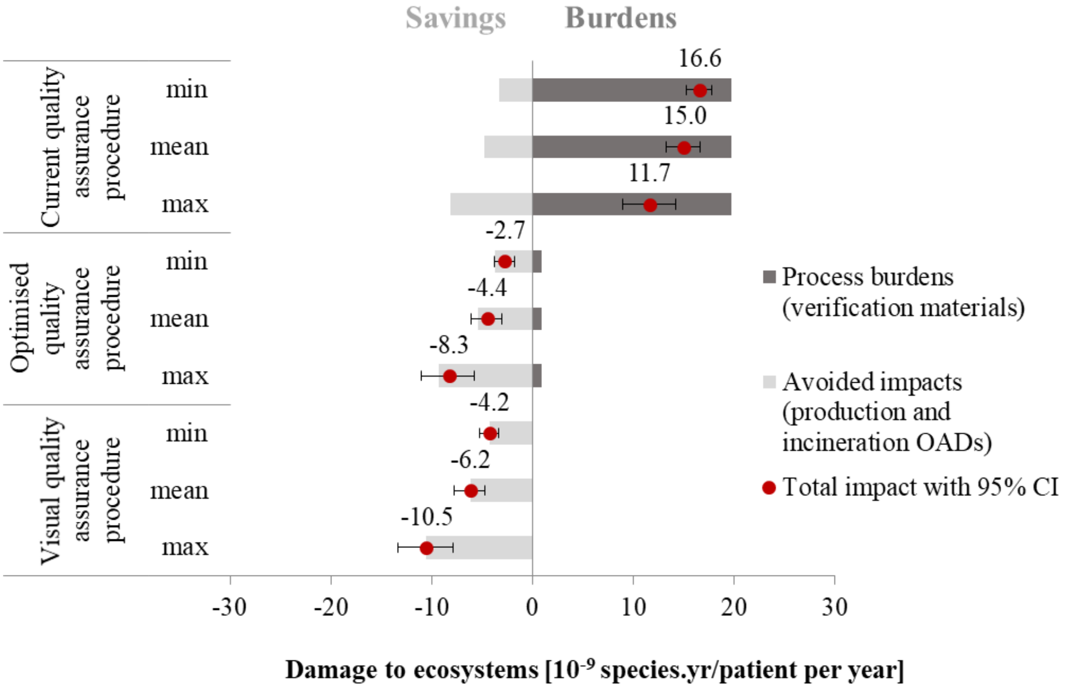

(C) Impacts on climate change

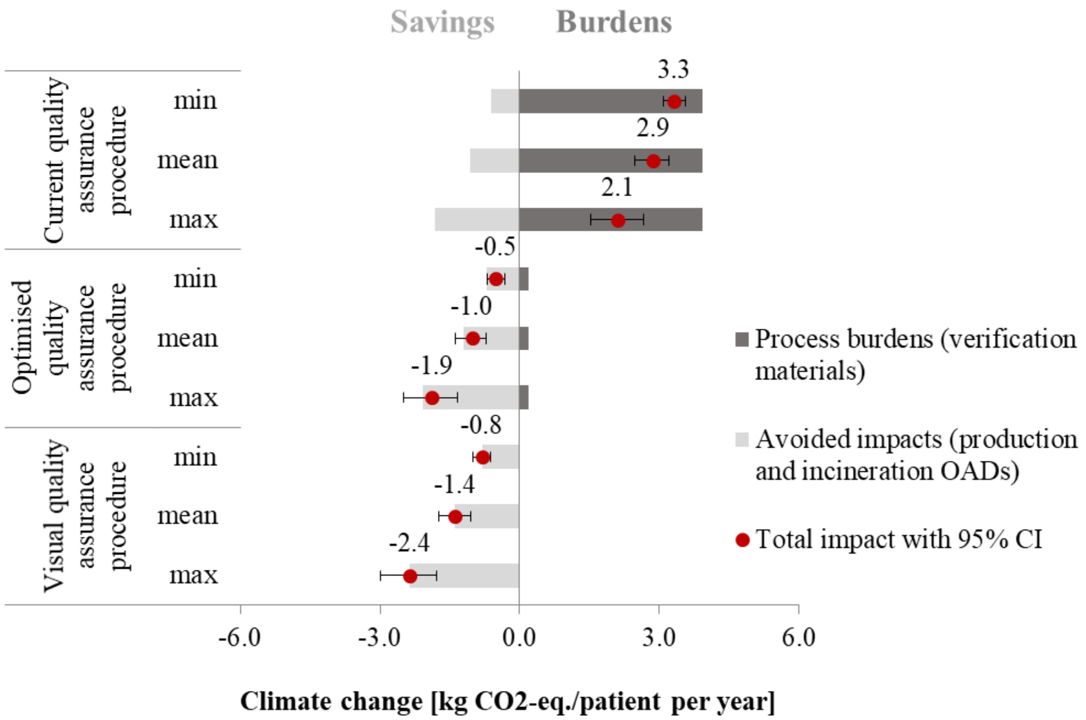

© 2024 Smale EM et al. *JAMA Network Open.*

eFigure 17. Results on OAD Production Level Including Solvent Recovery, Expressed per OAD Package and on API Production Level Expressed per g API on (A) & (B) Damage to Human Health, (C) & (E) Damage to Ecosystems and (D) & (F) Climate Change. This Scenario Lowered the Environmental Impacts of OAD Production With 49% for Damage to Human Health, 52% for Damage on Ecosystems, and 46% to Climate Change. Besides Lowering the Amount of Solvents Needed, Recycling Also Lowers the Volume That Needs to be Incinerated as Chemical Waste During Pharmaceutical Production. Solvent Recycling Results in Smaller Environmental Benefits for Redispensing, However, Environmental Benefits Are Still Obtained in the Optimized Quality Assurance Procedure and if the Quality Is Visually Checked.

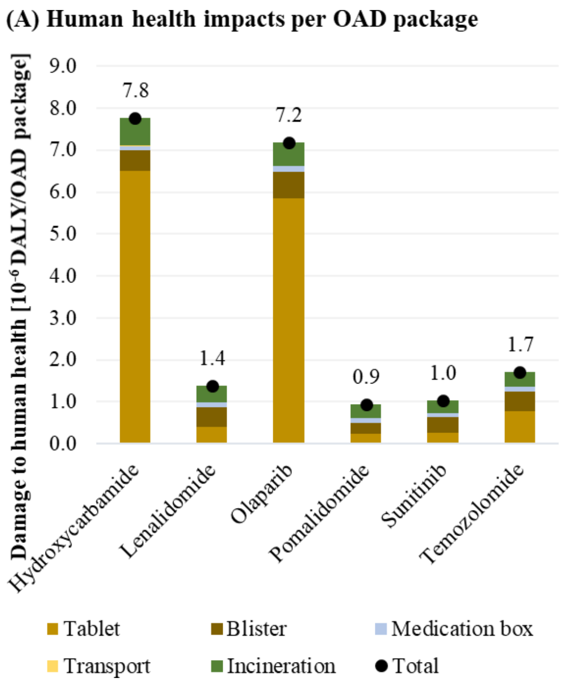

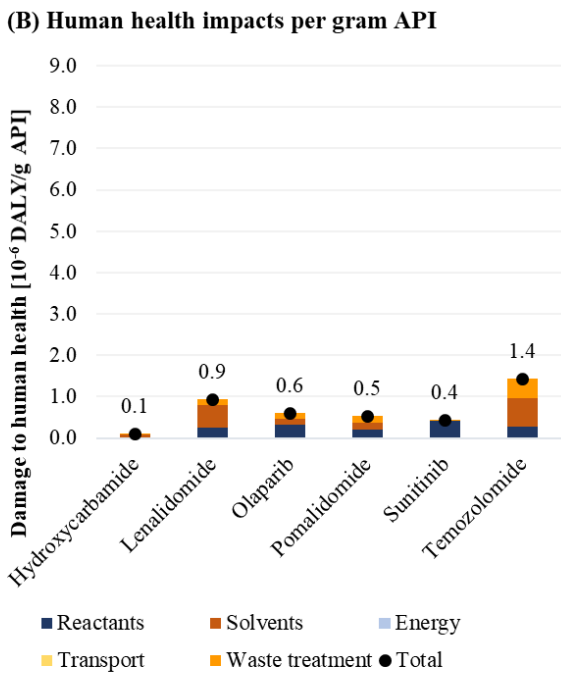

(C) Ecosystem impacts per OAD package

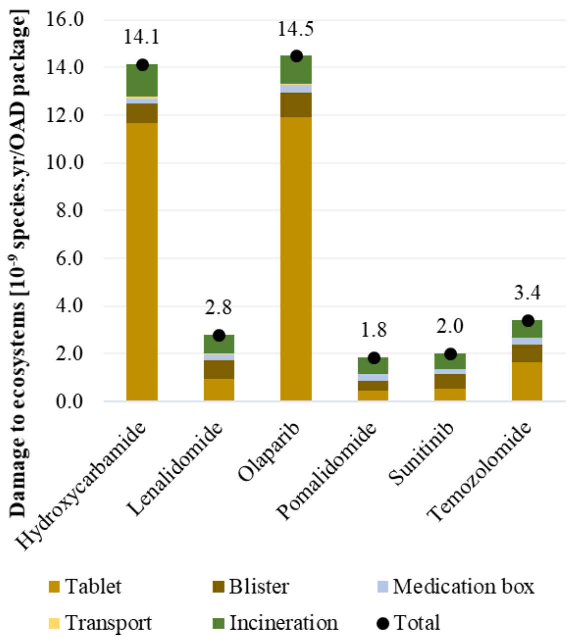

(D) Ecosystem impacts per gram API

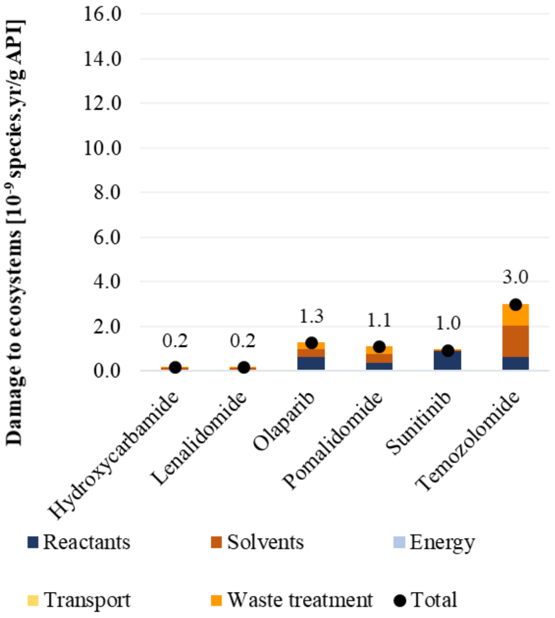

(E) Climate change impacts per OAD package

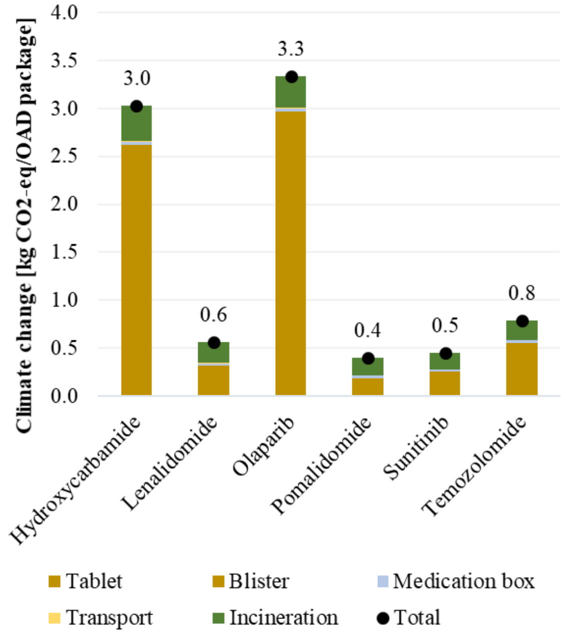

(F) Climate change impacts per gram API

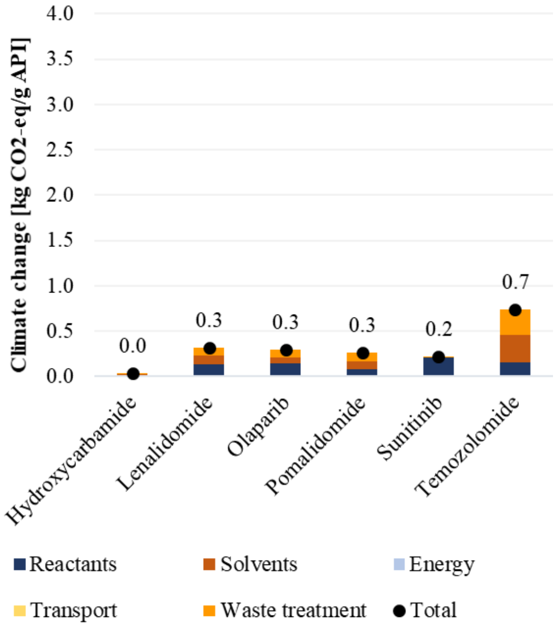

© 2024 Smale EM et al. *JAMA Network Open.*

eReferences.

1. Smale EM, van den Bemt BJF, Heerdink ER, et al. Cost Savings and Waste Reduction Through Redispensing Unused Oral Anticancer Drugs: The ROAD Study. *JAMA Oncol*. Published online 2023. doi:10.1001/jamaoncol.2023.4865

2. *Data to "Environmental Impacts of Reducing Medication Waste by Redispensing Unused Oral Anticancer Drugs: A Life Cycle Assessment."* doi:https://doi.org/10.17026/dans-zhs-q8et

3. Huijbregts M, Steinmann Z, Elshout P, et al. ReCiPe 2016 V1.1: A harmonized life cycle impact assessment method at midpoint and endpoint level Report I: Characterization. *ReCiPe 2016 Een geharmoniseerde levenscyclus impact Assess methode op "midpoint" en "endpoint" Niv Rapp 1 karakterisatie*. Published online 2017. https://rivm.openrepository.com/bitstream/10029/620793/3/2016-0104.pdf

4. Parvatker AG, Tunceroglu H, Sherman JD, et al. Cradle-to-Gate Greenhouse Gas Emissions for Twenty Anesthetic Active Pharmaceutical Ingredients Based on Process Scale-Up and Process Design Calculations. *ACS Sustain Chem Eng*. 2019;7(7):6580-6591. doi:10.1021/acssuschemeng.8b05473

5. Elsevier B.V. Reaxys - search substances, reactions, documents and bioactivity data. 2023. www.reaxys.com

6. Ecoinvent. EcoInvent database V3.8. Published online 2021. https://ecoquery.ecoinvent.org/3.8/apos/search

7. Wernet G, Bauer C, Steubing B, Reinhard J, Moreno-Ruiz E, Weidema B. The ecoinvent database version 3 (part I): overview and methodology. *Int J Life Cycle Assess*. 2016;21(9):1218-1230. doi:10.1007/s11367-016-1087-8

8. Piccinno F, Hischier R, Seeger S, Som C. From laboratory to industrial scale: a scale-up framework for chemical processes in life cycle assessment studies. *J Clean Prod*. 2016;135:1085-1097. doi:https://doi.org/10.1016/j.jclepro.2016.06.164

9. Constable DJC. Green and sustainable chemistry – The case for a systems-based, interdisciplinary approach. *iScience*. 2021;24(12):103489. doi:https://doi.org/10.1016/j.isci.2021.103489

10. United Nations Statistical Division. *International Stardard Industrial Classification of All Economic Activities*.; 2008.

11. Ecoinvent. *Ecoinvent 3 Report_transport Default Model_Global*.; 2022. https://ecoquery.ecoinvent.org/3.8/apos/search

12. The United States Pharmacopeial Convention. Geographic concentration of pharmaceutical manufacturing: USP Medicine Supply Map analysis. Accessed June 14, 2023. https://qualitymatters.usp.org/geographic-concentration-pharmaceutical-manufacturing

13. Wang D, Cheow WS, Amalina N, Faiezin M, Hadinoto K. Selecting optimal pharmaceutical excipient formulation from life cycle assessment perspectives: A case study on ibuprofen tablet formulations. *J Clean Prod*. 2021;292:126074. doi:https://doi.org/10.1016/j.jclepro.2021.126074

14. Ma Y, Zeng X, Ma X, Yang R, Zhao W. A simple and eco-friendly method of gelatin production from bone: One-step biocatalysis. *J Clean Prod*. 2019;209:916-926. doi:https://doi.org/10.1016/j.jclepro.2018.10.313

15. Akmalina R. Environmental impacts evaluation of sorbitol production from glucose. *Eksergi*. 2019;16(1):7-12.

© 2024 Smale EM et al. *JAMA Network Open.*
