# Supplementary material for: Environmental Outcomes of Reducing Medication Waste by Redispensing Unused Oral Anticancer Drugs
Source: JAMA Netw Open. 2024 Oct 10;7(10):e2438677. doi: 10.1001/jamanetworkopen.2024.38677 (PMC11581632; doi:10.1001/jamanetworkopen.2024.38677)
Supplement: Supplement 3. — Data Sharing Statement [file jamanetwopen-e2438677-s003.pdf]

Data Sharing Statement

Smale. Environmental Outcomes of Reducing Medication Waste by Redispensing Unused Oral Anticancer Drugs. *JAMA Netw Open*. Published October 10, 2024.
doi:10.1001/jamanetworkopen.2024.38677

Data
Data available: Yes
Data types: Deidentified participant data
How to access data: Charlotte.bekker@radboudumc.nl
When available: With publication

Supporting Documents
Document types: Statistical/analytic code, Other (please specify)
Additional Information: Inventory data tables
How to access documents: charlote.bekker@radboudumc.nl data repository:
https://doi.org/10.17026/dans-zhs-q8et
When available: With publication

Additional Information
Who can access the data: Researchers whose proposed use of the data has been approved.
Types of analyses: Research purposes
Mechanisms of data availability: With investigator support, after approval of a proposal.
